# Contrasting academic and lay press print coverage of the 2013-2016 Ebola Virus Disease outbreak

**DOI:** 10.1371/journal.pone.0179356

**Published:** 2017-06-22

**Authors:** Mark D. Kieh, Elim M. Cho, Ian A. Myles

**Affiliations:** 1 Laboratory of Clinical Infectious Diseases, National Institute of Allergy and Infectious Diseases, National Institutes of Health, Bethesda, Maryland, United States of America; 2 The George Washington School of Public Health MPH Program, Washington, DC, United States of America; Division of Clinical Research, UNITED STATES

## Abstract

Under a traditional paradigm, only those with the expected background knowledge consume academic literature. The lay press, as well as government and non-government agencies, play a complementary role of extracting findings of high interest or importance and translating them for general viewing. The need for accurate reporting and public advising is paramount when attempting to tackle epidemic outbreaks through behavior change. Yet, public trust in media outlets is at a historic low. The Crisis and Emergency Risk Communication (CERC) model for media reporting on public health emergencies was established in 2005 and has subsequently been used to analyze media reporting on outbreaks of influenza and measles as well as smoking habits and medication compliance. However, no media analysis had yet been performed on the 2013–2016 Ebola Virus Disease (EVD) outbreak. This study compared the EVD information relayed by lay press sources with general review articles in the academic literature through a mixed-methods analysis. These findings suggest that comprehensive review articles could not serve as a source to clarify and contextualize the uncertainties around the EVD outbreak, perhaps due to adherence to technical accuracy at the expense of clarity within the context of outbreak conditions. This finding does not imply inferiority of the academic literature, nor does it draw direct causation between confusion in review articles and public misunderstanding. Given the erosion of the barriers siloing academia, combined with the demands of today’s fast-paced media environment, contemporary researchers should realize that no study is outside the public forum and to therefore consider shifting the paradigm to take personal responsibility in the process of accurately translating their scientific words into public policy actions to best serve as a source of clarity.

## Introduction

Ebola virus disease (EVD) is a viral hemorrhagic disease that prior to 2013 was implicated in sporadic outbreaks in Equatorial Africa; the 2013–16 outbreak spread outside of Western Africa, and greatly heightened the lay press coverage of the disease. At the time, EVD treatment options were strictly supportive, leaving health officials with contact tracing, isolation, and behavior modification approaches to curtailing the outbreak [1]. The Crisis and Emergency Risk Communication (CERC) model for media reporting on public health emergencies was established in 2005 [2] and has subsequently been used to analyze media reporting on outbreaks of influenza [3, 4], measles [5], second hand smoke hazards [6], and medication adherence [7]. However, analysis has not been applied to the Western African outbreak of EVD despite the notable volume of media coverage and contemporaneous political debate. The need for accurate reporting and advising is paramount when attempting to tackle outbreaks through behavior change. However, today’s media environment carries the reputation of one more interested in speed to publication than accuracy of information–leading to the general public reporting a general lack of trust in the media [8] and creating the potential for the dissemination of dangerous misinformation. Completeness and accuracy are difficult to define in the fast moving world of scientific knowledge; sources viewed as correct reflections of a complete story one day may be viewed as half-truths the next. However, while it is beyond the scope of this project to attempt to provide the definitive story of EVD, it is possible to contrast the consensus information of the time with the contemporaneous reporting. This study aimed to provide novel insights into the completeness and accuracy of media reporting by applying the established CERC coding model to the 2013–2016 EVD outbreak across various levels of the written media–academic journals, news magazines, newspapers, Wikipedia, blogs, and alternative media outlets.

## Methods

### Article collection

**Fig 1:**
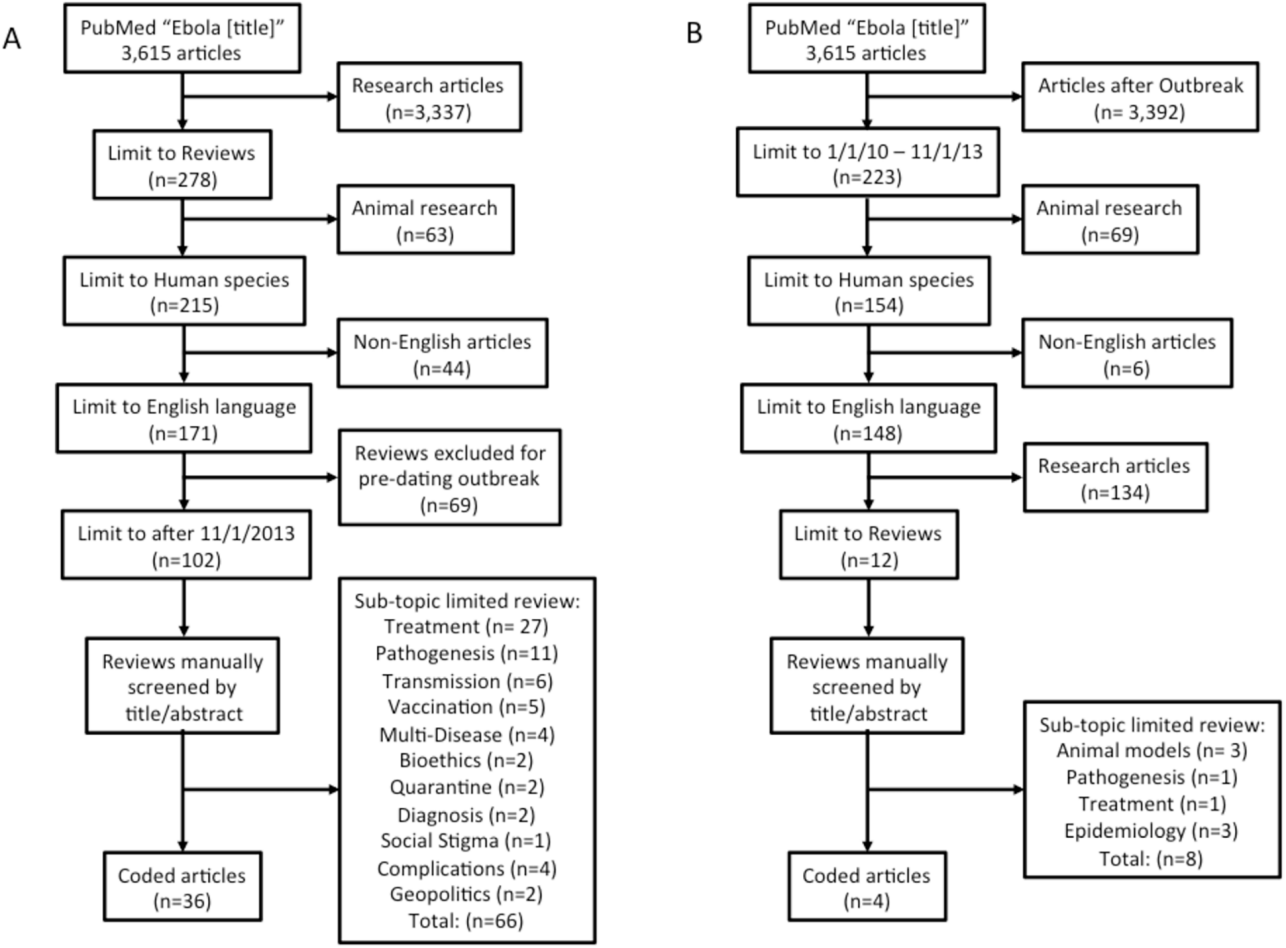
Systematic review inclusion process for academic articles focusing on general review. (A) The primary search for articles written after the Western African outbreak. (B) A similar search for general topic review articles from 2010 up until the Western African outbreak onset.

CERC modeling coding focused on the EVD outbreak with topics including prevention, trust and role of institutions, other, and unrelated information (Table 1). To select the academic papers a systematic literature review of PubMed articles was performed for maximal scope. Chosen articles contained “Ebola” in the title with further limitations including being an English-language review article, focused on humans, and published since 2013. Based on their abstract, coded academic articles were limited to review articles that aimed to provide an overview of the entire topic (such as “A primer on Ebola for clinicians” [9]) as compared to those reviewing a specific sub-topic (“Transmission dynamics and control of Ebola virus disease (EVD): a review” [10]; Fig 1). This search was repeated for articles dated from 2010-December 2013 to compare those written just prior to the 2013 outbreak. The first-search academic reference list can be found in the citations below [1, 9–109].

**Table 1:** The Crisis and Emergency Risk Communication (CERC) model for topic scoring. The CERC model was used to generate topic lists for scoring of accuracy, completeness, as well as enumerating unrelated information.

Code	Topic/Sub-Topic	Definition	Modifier	Example
**1**	**Ebola Outbreak**	Objective Information about the outbreak		
*1*.*1*	*Epidemiology*	Population statistics related to the outbreak	1.1.1	Number dead
			1.1.2	Number infected
			1.1.3	R' number (1.7–2)
			1.1.4	Fatality rate (16–92%)
	*Consequences*	What happens to those that contract Ebola		
*1*.*2*	Symptoms of Disease	Clinical presentation of patients with Ebola disease	1.2.1	Fever
			1.2.2	Headache
			1.2.3	Myalgias
			1.2.4	Arthralgias
			1.2.5	Abominal pain, nausea, vomiting, diarrhea
			1.2.6	Sore throat
			1.2.7	Oral ulcers
			1.2.8	Confusion
			1.2.9	Fatigue
			1.2.10	Loss of appetite
			1.2.11	Macular-Macolupapular rash
			1.2.12	Mucosal Hemorrhages
			1.2.13	Hiccups
*1*.*3*	*Laboratory abnormalities*	Objective lab measurements during Ebola disease	1.3.1	Leukopenia, lymphopenia, or leukocytosis
			1.3.2	Thrombocytopenia
			1.3.3	Transaminitis
			1.3.4	Hyperamylasemia
			1.3.5	Proteinuria
			1.3.6	Hypokalemia
			1.3.7	Lactic acidosis
			1.3.8	PT/PTT prolongation
			1.3.9	Decreased fibrinogen
			1.3.10	Viral RNA by PCR
			1.3.11	Viral Antibodies
**2**	**Perceived and Actual Risk**	How the public perceives risk of Ebola and risk factors for contracting Ebola		
*2*.*1*	*Vulnerable populations*	Populations with increased actual risk and severity for whom increased perceived risk is warranted	2.1.1	Pregnant females (abortion, placenta previa)
			2.1.2	Adults over 45
			2.1.3	Health care providers
			2.1.4	Breast feeding infants
*2*.*2*	*External factors*	Known environmental factors that increase the risk for Ebola	2.2.1	Mucosal-to-body fluid contact with infected individual (blood, urine, saliva, feces, emesis, breast milk, semen)
			2.2.2	Consumption of bush meat
			2.2.3	Funeral attendance
			2.2.4	Citizen of nation with poor health care system
			2.2.5	Citizen of nation with porous borders
			2.2.6	Living in area of high population density
			2.2.7	Citizen of nation with low trust in government or health institutions
			2.2.8	Deforestation/Vector & reservoir displacement
			2.2.9	Sex if virus present in semen
			2.2.10	Not airborne in nature
			2.2.11	Not infectious until symptomatic
*2*.*3*	*Incubation period*	Time from exposure to manifestation of symptoms within which concern for disease is valid		8-10d, never more than 21d
*2*.*4*	*Differential diagnosis*	Diseases that could mimic Ebola and thus impact perceived threat and severity due to false positives		Malaria, Typhoid, Traveler's diarrhea, Yellow fever
**3**	**Ebola Prevention**	Prevention measures taken to control the outbreak of Ebola		
*3*.*1*	*Primary Prevention (Biological)*	Therapeutic/pharmaceutical approaches to prevent contraction of Ebola		Ebola Vaccination
*3*.*2*	*Primary Prevention (Environmental)*	Environmental and behavioral modifications that can prevent contraction of Ebola	3.2.1	Contact and droplet precautions
			3.2.2	Isolation/Quarantine of suspected cases
			3.2.3	Contact tracing, monitoring 21 days
			3.2.4	Quick reporting/recognition of cases
			3.2.5	Class II biosafety handling of samples
			3.2.6	Frequent cleaning
			3.2.7	Behavior change communication/Community engagement
*3*.*3*	*Primary Prevention (PPE)*	Appropriate personal protective equipment worn by healthcare workers treated known/suspected cases of Ebola to prevent contraction of disease	3.3.1	Training donning and doffing
			3.3.2	Observed donning and doffing
			3.3.3	No exposed skin
			3.3.4	Impermeable gown
			3.3.5	Surgical mask
			3.3.6	Face shield
			3.3.7	Two sets of gloves
			3.3.8	N95 mask or Powered air purifying respirator (PAPR)
			3.3.9	Boots or shoe covers
			3.3.10	Waterproof apron/coverall
*3*.*4*	*Secondary prevention*	Treatment aimed at preventing disease consequences	3.4.1	Volume repletion
			3.4.2	Acid base balance
			3.4.3	Vasopressers
			3.4.4	Oxygen
			3.4.5	Pain control
			3.4.6	Nutrition
			3.4.7	Experimental therapies (Zmapp, Brincidofovir, convalescent serum)
			3.4.8	Supportive treatments are only option/no specific therapy
**4**	**Trust and role of institutions**	Trust in information supply		
*4*.*1*	*Nations impacted*	Nations impacted by Ebola as measured by one or more cases in their population/on their soil	4.1.1	Liberia
			4.1.2	Sierra Leone
			4.1.3	Guinea
			4.1.4	Nigeria
			4.1.5	Senegal
			4.1.6	Mali
			4.1.7	United States
			4.1.8	United Kingdom
			4.1.9	Italy/Germany
			4.1.10	Spain
*4*.*2*	*Agencies with expert knowledge/authority*	Referencing groups or individuals that are subject matter experts deserving trust on informing public	4.2.1	WHO/UN
			4.2.2	CDC
			4.2.3	MSF
			4.2.4	NIH
			4.2.5	Dept of Transportation
			4.2.6	Local Ministries of Health/Dept of health
			4.2.7	FDA
			4.2.8	Dept of Defense/USAMRIID
			4.2.9	USAID
			4.2.10	EPA/OSHA—PPE
			4.2.11	Red Cross
			4.2.12	HHS/ASPR/BRADA/USPHS
**5**	**Other**	Items not in categories above		
*5*.*1*	*Pathogenesis*	Information about how Ebola leads to morbidity or mortality		Inflammatory storm, sepsis, DIC, liver necrosis
*5*.*2*	*Virology*	Information about the virus that causes Ebola disease		Zaire strain, Filoviridae virus, ssRNA
*5*.*3*	*Animal Reservoir*	Non-human animals that can harbor and/or spread Ebola		Bats
**6**	**Historical Information**	Information that has nothing to do with current outbreak		Historical data on past outbreaks, information regarding nations involved, etc.
**7**	**Non-Factual statements**	Any claim that is inconsistent with the information above		
*7*.*1*	*Overt inaccuracies*	Any declarative claim that is inconsistent with the information above		"The outbreak began March 2014" stated after outbreak shown to have begun in December 2013
*7*.*2*	*Caveated inaccuracies*	Any statement that is factually inaccurate but stated as question of hearsay		"Some are asking if Ebola is spread through the air"
*7*.*3*	*Error by omission*	Any statement that could be misinterpreted due to lacking other pieces of key information		Providing only a partial list of PPE supplies Stating EVD airborne spread “can not be excluded” in context of outbreak conditions
*7*.*4*	*Claims without source/No evidence*	Statements made without appropriate citation		Academic journal citing LA times or opinion
**8**	**Unrelated**			
*8*.*1*	Medical Legal (hospital)	The legal requirements of the hospital		Did the hospital properly train their staff or was it allowable for them to release the names of infected nurses
*8*.*2*	Medical Legal (Individual)	The legal requirements of the individual		What are the rights under observation or quarantine
*8*.*3*	Celebrity donations	People providing money		Charities
*8*.*4*	Stigma	Treatment of survivors or nations afflicted as a whole		People pulling children from school
*8*.*5*	Geopolitics/Response/Geofinancing	How crisis impacts elections or how global decisions on funding are made		WHO payments, polling numbers, etc
*8*.*6*	Dem Republic of Congo	A concurrent outbreak occurred in DRC but was of a different and unrelated strain		Discussed but mentioned it was unrelated

Lay press sources were selected by viewership statistics. The top magazines, newspapers, and online blogs (as determined by subscription/traffic by The Alliance for Audited Media [110]) were searched using the same parameters as for the academic press. Top sources were: magazines (*AARP magazine*, *Cosmopolitan*, *Glamour*, *National Geographic*, *Time*); newspapers (*LA Times*, *Washington Post*, *New York Post*, *New York Times*, *USA Today*, *and Wall Street Journal*); blogs (*CBS*, *ABC/Yahoo*!, *NBC*, *CNN*, *Huffington Post*). Newspapers and magazines were indexed from LexisNexis Academic at Himmelfarb Library at the George Washington University Milken School of Public Health. Only one New York Post article was scored since the remaining were behind a prohibitive pay-wall. Blog sites may have differing algorithms for their internal searches, all blog posts were thus collected by using the Google function of “site:blog name.com ebola[title]”. Alternative sites (InfoWars, VeteransToday, Beforeitsnews, NewsBusters, and NoDisinfo) were selected after having been identified as the top five sites of social influence by recent media assessments [111]. Top pages from blogs and alternative sites were selected based on Google’s relevance assessment. Lay press search yielded 38 magazine, 219 newspaper, and 29,850 blog posts. Time and resources precluded scoring of all blog posts and thus, only the top 30 for each group were read. After eliminating duplicates (for example when *ABC/Yahoo*! carried a *Huffington Post* article) a total of 133 were scored as a representative sample (S1 Appendix). Wikipedia articles were collected by downloading the EVD page on the same date that blog and newspaper searchers were performed. Prior versions were accessed within the Wikipedia site and the page status immediately prior to the December 2013 outbreak was taken as a pre-outbreak comparator. Potential covariates were documented such as date of publication, copy length, author’s nation of origin, total pages, impact factor, readership, number of authors, and citations.

### Coding

All articles were printed, scores were written in the margins for each article and later entered into an Excel spreadsheet (S1 Table). Two individuals scored each article; one author (IM) scored all articles while the second reading was divided among research assistants (MK, EC) who scored a clean copy of the article to assure they were not influenced by the prior scoring. Inter-reader variation was calculated by taking the total number of topics and subtopics scored for agreement (110, plus additional error scores if more than one error of each type was found), subtracting the number of discordant topic points (topics that primary reader gave credit but the secondary reader did not or vice versa), then dividing by the total possible points. Scores were compared across readers and ranged from 95.4–100% inter-reader agreement. All discordant points were evaluated by the team, resolved to consensus, and reported.

### Error and completeness determinations

Articles were scored for completeness on general themes of commentary. For example, statements of “elevated temperature” were scored as mentioning “fever” so that scoring was offset in favor of credit for mentioning topics. However, sweeping statements were not broadly scored. For example, mentioning EVD spread “outside of Western Africa” did not entail credit for specific nations involved. Similarly, statements discussing personal protective equipment were not credited unless the specific item was named.

As much as possible, each article was judged as if it each was the only one available to the reader. Therefore, stating that personal protective equipment (PPE) entailed “gloves, gown, and face mask” was considered an error of omission given that a reader would not know additional PPE would be needed unless they had read additional sources. Overt errors were statements that were simply untrue, such as claims that the outbreak began in March of 2014 instead of December 2013 [86] (which would not have been erroneous if stated in the early stages of the outbreak, but this information was established by the time of the article’s publication). Caveated inaccuracies were those presenting inaccurate statements as uncertainty (such as “Airborne spread is rare or absent” [14] when only “absent” is correct) or to place an unnecessary qualifier in front of accurate statements that has the potential to cast doubt (such as “from what we know of the science, Ebola is not an airborne disease”)(Huffington Post 11)). Such qualifiers placed in front of discussion of airborne transmission, but not in front of any other statement could suggest doubt (for example, no article stated ‘from what we know, EVD is a viral disease’). The final error category was statements made without citation to a verifiable source.

### Statistics

Each article was scored for all topics and transcribed into a central scorecard (S1 Table). The mean calculated the average completeness and inaccuracies for each category of written media were compared by ANOVA. Linear regression was calculated for all categories as determined by PRISM GraphPad analytic and graphing software (La Jolla, CA). Logistic regressions was performed for inaccuracy and completeness versus the covariates listed.

## Results

### EVD academic articles had a consensus narrative

Based on the articles reviewed [1, 9, 14, 15, 18, 23, 26, 31, 36, 38, 45, 59–61, 64, 68, 69, 71, 74, 78, 79, 83, 85, 86, 89, 92, 93, 97, 99, 105, 112–116] a consensus narrative could be constructed for the EVD outbreak (see Table 2, S1 Table for details). It is important to note that we do not attempt to present the narrative as more than what can be gleaned from the collection of scored review articles. This narrative should therefore not serve as a historical source document given that it lacks of historical accounts that fell outside the purview of the academic reviews.

**Table 2:** The resultant scores for comprehensive review articles under Crisis and Emergency Risk Communication (CERC) model. Scores for presence or absence of covered topics were generated using the CERC model in Table 1. Citations 111–114 pre-date 2013 EVD outbreak. Red indicates the citation covered the topic, blue indicates the topic was not covered, and grey indicates not applicable.

Citation	112	113	114	115	1	9	14	15	18	23	26	31	36	38	45	55–58	59	60	61	64	68	69	71	74	78	79	83	85	86	89	92	93	97	99	105	116
Number dead																																				
Number infected																																				
R' number (1.9–4.1)																																				
Fatality rate (16–92%)																																				
Fever																																				
Headache																																				
Myalgias																																				
Arthralgias																																				
Abdominal pain, nausea, vomiting, diarrhea																																				
Sore throat/Oral ulcers																																				
Hypotenion/Shock, Multi-organ/system failure																																				
Neurologic/Confusion																																				
Fatigue/Malasie																																				
Shortness of breath, chest pain, cough																																				
Macular-Macolupapular rash																																				
Mucosal Hemmorrhages																																				
Hiccups																																				
Leukopenia, lymphopenia, or leukocytosis																																				
Thrombocytopenia																																				
Transaminitis/Hepatitis																																				
Hyperamylasemia/Pancreatitis																																				
Proteinuria/Edema/low albumin																																				
Electrolyte abnormalities/renal dysfunction																																				
Lactic acidosis																																				
PT/PTT prolongation/Coagulopathy																																				
Decreased fibrinogen/DIC																																				
Viral RNA by PCR																																				
Viral Antibodies																																				
Pregnant females (abortion, placenta previa)																																				
Adults over 45																																				
Health workers																																				
Breastfeeding infants																																				
Mucosal-to-body fluid contact																																				
Consumption of bush meat (rat, primate)																																				
Unsafe burial practices																																				
Citizen of nation with poor health care system																																				
Citizen of nation with porous borders																																				
Living in area of high population density																																				
Low trust in government or institutions																																				
Deforestation/Vector & resivour displacement																																				
Sex if virus present in semen																																				
Not airborne in nature																																				
Not infectious until symptomatic																																				
*Incubation period (2-21d)*																																				
*Differenital diagnosis*																																				
*Primary Prevention (Biological)*																																				
Contact and droplet precaustions																																				
Isolation/Quarantine of suspected cases																																				
Contact tracing, monitoring																																				
Quick reporting/recognition of cases																																				
Class II/BSL4 biosafety handling of samples																																				
Environmental decontamination																																				
Behavior change comm/Community engagement																																				
Training donning and doffing																																				
Observed donning and doffing																																				
No exposed skin																																				
Impermiable gown																																				
Surgical mask																																				
Face shield																																				
Two sets of gloves																																				
N95 mask or PAPR																																				
Boots or shoe covers																																				
Waterproof apron/coverall																																				
Volume repletion																																				
Acid base balance																																				
Vasopressers																																				
Oxygen																																				
Pain control																																				
Nutrition																																				
Experimental therapies																																				
Supportive treatment only																																				
Travel bans (not helpful)																																				
Screening Travelers																																				
Liberia																																				
Sierra Leone																																				
Guinea																																				
Nigeria																																				
Senegal																																				
Mali																																				
United States																																				
United Kingdom																																				
Italy/Germany																																				
Spain																																				
WHO/UN																																				
CDC																																				
MSF																																				
NIH																																				
Dept of Transportation																																				
Local Ministries of Health/Dept of Health																																				
FDA																																				
Dept of Defense/USAMRIID																																				
USAID																																				
EPA/OSHA																																				
Red Cross																																				
HHS/ASPR/BRADA/USPHS																																				
*Molecular/Cellular Pathogenesis*																																				
*Virology*																																				
*Animal Resivour*																																				
Historical Perspective																																				
*Overt inaccuracies*																																				
*Caveated inaccuracies*																																				
*Error by omission*																																				
*Uncited claim/No evidence*																																				

Ebola viruses were discovered in 1976 during an outbreak in Zaire (which is now the Democratic Republic of the Congo) and an outbreak in what is now South Sudan. There have been sporadic outbreaks since then, some of which have taken place in Uganda. The outbreak of EVD beginning in December 2013 thrust the disease into the forefront of both media and science through claiming the lives of at least 11,325, infecting at least 28,657, and carrying a fatality rate of approximately 40%. The nations most impacted were Liberia, Guinea, and Sierra Leone, but disease also spread to Nigeria, Senegal, and Mali. Unlike prior outbreaks, EVD affected nations outside of the African continent: the US, UK, Italy, Germany, and Spain all had citizens either infected or treated on their soil. Responding to the crisis involved extensive coordination between multiple international agencies.

The selected review articles agreed that the initial responders were the Guinean Ministry of Health, Médecins Sans Frontières (MSF; Doctors Without Borders), the World Health Organization (WHO) and the US Center for Disease Control (CDC). However, the review articles presented conflicting accounts of the exact timing and coordination of the agencies respective involvements. The National Institutes of Health (NIH), the Food and Drug Administration (FDA), along with other international groups worked on the development and testing of vaccines and therapies that had been in development for decades but lacked the needed funding, priority, and clinical opportunities to enter clinical trials. The Environmental Protection Agency (EPA), Occupational Safety and Health Administration (OSHA), and the Department of Transportation were in charge of assuring safe transport and testing of patients, blood samples, and contaminated supplies within the US. When the death toll in Liberia threatened to enter exponential growth, President Obama called for military aide through the Department of Defense to bolster the diagnostic support being provided by USAMRIID (US Army Medical Research Institute in Infectious Disease); the Defense Department built an EVD treatment unit (ETU) in Liberia while members of the US Public Health Service Commissioned Corps, the uniformed service under the Surgeon General and the Department of Health and Human Services staffed this ETU. Their assistance joined groups already on the ground like the Red Cross, USAID, and many other smaller charities, faith-groups, and agencies from around the world.

Ebola virus is one of the five known ebolaviruses (RNA viruses of the family Filvovirus). Four ebolaviruses (Sudan virus, Bundibugyo virus, Tai Forest virus, and Ebola virus) cause EVD, whereas Reston virus only infects non-human primates and pigs. While EVD is one of the hemorrhagic fevers (along with disease caused by Marburg virus and others), in contrast to pop-culture imagery less than half [117] of the patients stricken with EVD manifest bleeding complications. Bleeding is a negative prognostic indicator and, when present, indicates late stage disease. Therefore its absence should not deter from the diagnosis, especially when the patient may be early in the disease course. Gastrointestinal symptoms are also common and include abdominal pain, diarrhea, anorexia, nausea, and vomiting. Additional potential symptoms include headache, muscle and joint aches, sore throat, confusion, fatigue, shortness of breath, chest pain, and occasionally a maculopapular rash. Hiccups are an established symptom; while not universal, they may help to distinguish EVD and Marburg infection [118] from those disorders with similar presentations such as malaria and Dengue.

The pathogenesis of EVD begins when the virus enters the body after direct contact of infected body fluid with a mucosal surface. Ebola virus is taken up by antigen-presenting cells (APC) like macrophages and dendritic cells, which then migrate to the lymph nodes where they would normally help amplify the normal immune response. However in EVD, the APC die and spread the virus into the lymphatic system and eventually the spleen. As immune cells become infected, they release inflammatory proteins called cytokines, which attract other immune cells to the infected area that are then also infected. This process amplifies upon itself, cells become infected, die, produce cytokines, bringing in more cells, which become infected, die, etc. This creates an uncontrolled cascade of inflammation known as a “cytokine storm” which can damage the patient’s own tissues and organs. Furthermore, as Ebola virus kills immune cells, the body is left susceptible to other forms of infection. In fatal cases, infection and tissue damage cause death through shock and multisystem organ failure.

The preferred laboratory test for EVD is a viral RNA level by PCR, however viral antibodies are also used as a diagnostic tests. EVD may cause abnormalities in white blood cell counts (either too many or too few), decreased platelets, along with evidence of hepatitis, renal failure, protein loss, and pancreatitis. Advanced disease is associated with lactic acidosis, clotting abnormalities, and evidence of disseminated intravascular coagulopathy. If bleeding does occur, it is due to both this disrupted clotting system and profound inflammation in mucosal tissues allowing blood to leak through the normal barriers.

Because EVD is only contracted through direct contact with body fluids, health care workers are particularly at risk, even if only the smallest patch of skin exposed. The supplies for full PPE are extensive and include impermeable gowns or more commonly coveralls, surgical masks, face shields, two sets of gloves, boots, a waterproof apron, with mouth and nose coverings, that may include an N95 respirator. However, extensive training in putting on and taking off the PPE (termed donning and doffing) is required along with a trained observer of both of these processes. The intricacies of PPE training are complex and considered beyond the scope of this manuscript.

Patient care for EVD is primarily volume replacement, pain control, acid/base balancing, and support for blood pressure, oxygen, and nutrition. While experimental therapies, and a handful of vaccination strategies showed promising results during the 2013–2016 outbreak, they did not directly contribute to ultimately controlling the disease. Rather standard public health measures were able to curtail the outbreak such as rapid identification and isolation of confirmed cases followed by tracing all their contacts and daily monitoring and/or quarantining them for the recommended 21-days. While the CDC and WHO supported screening travelers for fever departing from Western African nations and upon final arrival, a blanket travel ban was not advised. Travel bans have been counter-productive in past pandemics, as they blocked needed resources from entering and leaving afflicted nations. While recommendations for handling samples in a biosafety level 4 (BSL-4) facility was not fully achievable in the Western African environment, strict and frequent decontamination of sample processing and patient care areas with bleach containing solutions was widely practiced.

In addition to health providers, pregnant women and breast-feeding infants are also at particular risk for complications and transmission of the disease. Children and adults over 45 are populations that are less likely to contract EVD, however these groups also have an increased risk for fatal outcomes. Individuals with EVD are not contagious until viral loads in the blood are high enough to cause shedding and are not infectious until after they have begun manifesting symptoms.

Simply put, EVD cannot be spread from human-to-human in the air during outbreak conditions. Some confusion may have arisen given that “droplet spread” can occur by sneezing, which does transmit ‘through air’. However, airborne spread is defined by far greater distances of spread and often a far greater infectivity as measured by an R_0_ (R-naught) number. The R_0_ for EVD (1.9–4.1) is much closer to other fluid-borne diseases like HIV (4) than airborne disease like measles (>18). Effectively, if you are not within close proximity (approximately two meters, or six feet) [119] of a person who has had active signs of EVD, you do not have to fear catching the disease. This rule was somewhat shaken when it was discovered that female sexual partners of male EVD survivors could contract the disease due to viral persistence in semen for several months. Confusing word choice may be to blame for some of the misconceptions surrounding when a person is contagious. Some outlets stated that you could not contract EVD from a person *unless* they were symptomatic. This was communicated to assure the public of the fact that travelers that passed fever screenings upon entering the US would not be contagious to any of their fellow passengers. A better phrasing may be that a person is not contagious *until* they manifest symptoms; this phrasing would capture the potential for survivors to spread the virus via semen after active symptoms had resolved. However, these semantics would not impact the consensus that patients manifesting symptoms of EVD are routinely too sick to perform activities of daily living like shopping, driving, or riding public transportation.

Viral loads increase as the severity of the disease progresses and therefore the bodies of the recently deceased are particularly dangerous. Burial practices in Western Africa included a tradition of kissing and washing dead bodies as a family; this practice was an important source of EVD transmission during the 2013–2016 outbreak. Many in the Western African regions, especially during the early portion of the outbreak, refused to alert officials to potential EVD cases in their families. Years of mistrust in government and foreigners led to initial conflict between the population and the care providing institutions. Additional population risk factors that made Western Africa susceptible for an EVD outbreak included densely populated cities, porous borders, and the profound poverty and under resourced health care systems of the region. A few sources postulated that food insecurity due to climate-change is also theorized to have spurred the outbreak as it increased the need for reliance on the consumption of ‘bush meat’ that could have come from infected animals [26, 68]. These authors also implicated deforestation as a possible contributor to the outbreak by increasing the interaction of humans with the newly displaced animal population, particularly bats, which are presumed to be the animal vectors for EVD [26, 68]. However, most articles did not cite these environmental findings as underpinnings of the outbreak.

### The 2013 EVD outbreak greatly increased academic journal attention

Between 2010 and December 2013 there were 150 total and 15 English review articles focusing on human disease. From 2013 through the search date, there were 3,615 total articles and 278 review articles (Fig 1). This represents a nearly 25-fold increase in annual academic coverage. There were only 4 general review articles between 2010–2013 compared to 32 after the onset of the Western African outbreak (4 of the 36 post-outbreak articles were intended to be read as a special issue and were thus scored as an aggregate). Authors of the pre-outbreak general review articles were either from the United States (two articles), Tanzania, or Gabon. Post-outbreak saw a great geographic expansion to include Canada, India, Uganda, the United Kingdom, China, France, Gambia, Saudi Arabia, Sweden, Turkey, Poland, and the United States.

### Qualitative results

#### Journals themes expanded to focus on treatment and prevention after the outbreak onset

Within the review articles, while most of the scored topics saw a decreased frequency of representation after the 2013 outbreak began (Table 3), there were several topics that received much greater attention after 2013. Review articles were far more likely to discuss elements needed for treatment and containment after the outbreak. For example, mentioning of contact tracing and isolation, detailing the recommended PPE, and the treatment recommendations were all more likely to appear after 2013. It is worth noting that the treatment and containment topics did not contain new information; this information garnered increased focus but did not differ from pre-2013 manuscripts as to reflect ‘lessons learned’ during the outbreak. Topics that did appear *de novo* after 2013 included the increased risk of spontaneous abortion for pregnant females and the established finding that hiccups may be a manifestation of EVD, which was overlooked by the review articles selected. The hiccup finding is not a trivial one; it was reported that MSF received early reports of patients with viral syndromes accompanied by hiccups [17]. While this is not a pathognomonic finding, a clustering of suspicious deaths, which included the presence of hiccups alerted MSF officials that the outbreak potentially represented a serious outbreak of hemorrhagic fever. This was the basis for their early appeals to the World Health Organization (WHO) to provide a rapid response to the first EVD outbreak in a densely populated area [17].

**Table 3:** Completeness and accuracy scores by media type. Aggregate scores for coverage of topics were collected for each media type based on individual media outlets (S1 Table). Nations impacted in the 2013–2016 outbreak were not counted against those sources that pre-dated the outbreak.

	Journals		Wikipedia		Magazines	Newspapers	Blogs	Alternative Sites
Topic/Sub-Topic	Pre-Outbreak	Post-Outbreak	Pre-Outbreak	Post-Outbreak				
**Ebola Outbreak**								
*Epidemiology*								
Number dead	100	84.4	100	100	28.9	36.0	71.4	19.2
Number infected	100	81.3	100	100	36.8	20.7	43.6	7.7
R' number (1.9–4.1)	0	21.9	0	0	0.0	0.3	0.0	0
Fatality rate (16–92%)	100	71.9	100	100	15.8	7.0	12.0	3.8
*Symptoms of Disease*								
Fever	100	90.6	100	100	31.6	22.3	15.0	7.7
Headache	75	75.0	100	100	13.2	2.3	3.0	3.8
Myalgia	100	62.5	100	100	10.5	2.7	2.3	3.8
Arthralgia	25	18.8	0	100	7.9	1.0	1.5	0
Abdominal pain, n/v, diarrhea	100	90.6	100	100	23.7	12.3	9.8	0
Sore throat/Oral ulcers	25	28.1	0	100	7.9	0.7	2.3	3.8
Hypotension/Shock, Multi-organ/system failure	75	65.6	100	100	5.3	1.0	2.3	0
Neurologic/Confusion	100	31.3	100	100	2.6	0.3	0.8	0
Fatigue/Malaise	75	71.9	100	100	18.4	2.0	5.3	0
Shortness of breath, chest pain, cough	100	56.3	100	100	0.0	1.3	0.8	3.8
Macular-Macolupapular rash	75	53.1	100	100	0.0	0.3	1.5	0
Mucosal Hemorrhages	100	90.6	100	100	10.5	6.3	6.0	7.7
Hiccups	0	28.1	100	0	2.6	0.0	0.0	0
*Laboratory abnormalities*								
Leukopenia, lymphopenia, or leukocytosis	50	46.9	0	100	0.0	0.0	0.0	0
Thrombocytopenia	50	43.8	0	100	0.0	0.3	0.0	0
Transaminitis/Hepatitis	50	53.1	0	100	0.0	0.3	1.5	0
Hyperamylasemia/Pancreatitis	0	21.9	0	0	0.0	0.0	0.0	0
Proteinuria/Edema/low albumin	50	31.3	0	100	0.0	0.0	0.0	0
Electrolyte abnormalities/renal dysfunction	0	59.4	0	100	5.3	1.7	3.8	0
Lactic acidosis	0	15.6	0	0	0.0	0.0	0.0	0
PT/PTT prolongation/Coagulopathy	75	37.5	100	100	2.6	0.0	0.8	0
Decreased fibrinogen/DIC	75	62.5	100	100	0.0	0.0	0.0	0
Viral RNA by PCR	75	71.9	0	100	0.0	0.7	2.3	7.7
Viral Antibodies	75	75.0	100	100	0.0	0.3	0.8	0
**Perceived and Actual Risk**								
*Vulnerable populations*								
Pregnant females (abortion, placenta previa)	0	15.6	0	100	2.6	0.3	0.0	0
Adults over 45	0	6.3	0	0	0.0	0.0	0.8	0
Health workers	75	62.5	0	100	42.1	14.3	19.5	3.8
Breastfeeding infants	0	12.5	0	100	2.6	0.3	1.5	0
*External factors*								
Mucosal-to-body fluid contact	50	87.5	100	100	26.3	15.3	24.1	7.7
Consumption of bush meat (rat, primate)	50	59.4	100	100	7.9	2.0	3.0	0
Unsafe burial practices	50	68.8	100	100	7.9	4.7	11.3	3.8
Citizen of nation with poor health care system	50	56.3	100	100	28.9	12.7	12.8	0
Citizen of nation with porous borders	0	25.0	0	0	5.3	1.7	3.0	0
Living in area of high population density	0	34.4	0	0	5.3	1.3	0.8	0
Low trust in government or institutions	50	34.4	0	100	15.8	3.0	7.5	3.8
Deforestation/Vector & reservoir displacement	25	6.3	0	0	2.6	0.3	0.0	0
Sex if virus present in semen	75	21.9	100	100	2.6	2.0	10.5	0
Not airborne in nature	50	65.6	100	100	18.4	4.0	4.5	0
Not infectious until symptomatic	0	28.1	0	100	31.6	7.7	9.0	3.8
*Incubation period (2-21d)*	75	81.3	100	100	21.1	20.0	21.1	23.1
*Differential diagnosis*	50	43.8	100	100	13.2	1.3	3.0	3.8
**Ebola Prevention**								
*Primary Prevention (Biological)*	75	68.8	100	100	15.8	8.3	19.5	23.1
*Primary Prevention (Environmental)*								
Contact and droplet precautions	0	15.6	0	0	0.0	0.0	0.0	0
Isolation/Quarantine of suspected cases	25	56.3	100	100	28.9	33.3	23.3	19.2
Contact tracing, monitoring	25	50.0	0	100	18.4	16.7	27.1	0
Quick reporting/recognition of cases	25	59.4	0	100	7.9	4.0	2.3	3.8
Class II/BSL4 biosafety handling of samples	75	34.4	100	100	5.3	0.3	3.0	3.8
Environmental decontamination	50	43.8	100	100	18.4	6.7	13.5	11.5
Behavior change comm/Community engagement	25	18.8	100	100	5.3	4.3	6.8	3.8
*Primary Prevention (PPE)*								
Training donning and doffing	0	40.6	100	100	7.9	6.0	1.5	3.8
Observed donning and doffing	0	31.3	0	100	2.6	3.3	0.8	0
No exposed skin	0	25.0	0	100	7.9	2.7	0.8	0
Impermeable gown	50	46.9	100	100	7.9	4.7	3.8	0
Surgical mask	50	31.3	100	100	7.9	4.0	3.8	0
Face shield	50	43.8	100	100	7.9	4.0	1.5	0
Two sets of gloves	50	46.9	0	100	5.3	5.3	5.3	0
N95 mask or PAPR	0	34.4	0	100	0.0	0.3	0.0	0
Boots or shoe covers	0	40.6	0	100	5.3	1.7	0.0	0
Waterproof apron/coverall	0	31.3	0	100	2.6	1.0	0.0	0
*Secondary prevention*								
Volume repletion	25	65.6	100	100	15.8	6.0	3.8	0
Acid base balance	0	46.9	100	100	10.5	0.3	0.0	0
Vasopressers	0	25.0	0	0	5.3	0.7	0.8	0
Oxygen	0	12.5	100	100	2.6	2.0	1.5	0
Pain control	0	28.1	100	100	0.0	0.3	1.5	0
Nutrition	0	12.5	0	0	0.0	0.0	0.0	0
Experimental therapies	100	84.4	100	0	18.4	16.7	20.3	0
Supportive treatment only	75	84.4	100	100	13.2	8.7	2.3	0
*Preventing spread*								
Travel bans (not helpful)	0	0.0	0	100	5.3	4.7	3.0	3.8
Screening Travelers	0	56.3	0	100	13.2	14.0	4.5	15.4
**Trust and role of institutions**								
*Nations impacted*								
Liberia		93.8		100	57.9	57.0	69.9	42.3
Sierra Leone		93.8		100	36.8	42.7	69.2	19.2
Guinea		93.8		100	28.9	40.7	68.4	15.4
Nigeria		78.1		0	23.7	9.0	6.8	3.8
Senegal		65.6		0	15.8	2.7	4.5	3.8
Mali		21.9		0	2.6	1.7	3.0	0
United States		65.6		100	57.9	38.3	27.8	76.9
United Kingdom		6.3		100	5.3	1.0	7.5	0
Italy/Germany		3.1		0	0.0	1.0	0.8	0
Spain		46.9		100	15.8	7.0	8.3	0
*Agencies*								
WHO/UN	0	84.4	100	100	21.1	44.0	59.4	19.2
CDC	25	53.1	100	100	42.1	41.3	30.1	38.5
MSF	25	21.9	100	100	18.4	15.0	20.3	0
NIH	0	18.8	0	0	5.3	12.0	8.3	7.7
Dept of Transportation	0	3.1	0	0	2.6	0.3	0.0	0
Local Ministries of Health/Dept of health	0	9.4	100	0	5.3	13.0	17.3	0
FDA	25	34.4	100	100	2.6	7.0	7.5	7.7
Dept of Defense/USAMRIID	0	6.3	100	100	13.2	13.3	7.5	26.9
USAID	0	3.1	0	0	5.3	3.7	2.3	0
EPA/OSHA—PPE	0	12.5	0	0	0.0	0.3	0.0	0
Red Cross	0	3.1	0	0	7.9	3.0	4.5	0
HHS/ASPR/BRADA/USPHS	0	6.3	0	0	2.6	5.7	0.8	3.8
**Other**								
*Molecular/Cellular Pathogenesis*	75	68.8	100	100	2.6	0.3	3.0	0
*Virology*	100	90.6	100	100	5.3	0.0	0.8	3.8
*Animal Reservoir*	50	81.3	100	100	7.9	3.0	6.8	0
Historical Perspective	100	68.8	100	100	7.9	1.0	3.0	3.8
**Non-Factual statements (% of articles with)**								
*Overt inaccuracies*	0	9.4	100	0	0.0	3.3	1.5	61.5
*Caveated inaccuracies*	0	3.1	0	0	0.0	1.0	0.8	0
*Error by omission*	0	34.4	0	0	5.3	3.7	1.5	0
*Claim without source or evidence*	0	3.1	0	0	0.0	0.0	0.0	19.2
**Unrelated**								
Medical Legal (hospital)	0	0.0	0	0	2.6	4.7	4.5	11.5
Medical Legal (Individual)	0	0.0	0	0	5.3	2.7	0.0	7.7
Celebrity donations	0	0.0	0	0	13.2	0.3	1.5	0
Stigma	0	0.0	0	0	26.3	8.7	12.8	0
Geopolitics/Response/Geofinancing	0	0.0	0	0	5.3	29.0	21.1	42.3
Dem Republic of Congo	0	0.0	0	0	0.0	1.7	1.5	0

#### The academic press reviewed for the study did not cover behavior change or the local ministry of health

Academic journal articles were highly focused on the need for developing a vaccine or novel therapeutic to curtail the outbreak. Many went as far as to claim that the outbreak would not be contained without such discoveries [34, 36, 64, 68, 85]. However, the outbreak was controlled by standard methods of public health and behavior change communication (BCC) that even caused some modifications of vaccine studies due to a lack of background infection rates [120] (WSJ 241, 249, 257; CBS 26; Yahoo!/ABC 13; CNN 5). Even academic press published late in the outbreak’s time course did not detail the impact that non-pharmaceutical interventions had in infection control, nor did they discuss the need for international coordination with the local Ministry of Health within the afflicted region. In fact, a smaller percentage of articles mentioned these non-pharmaceutical interventions after the outbreak than before.

#### Qualitative themes differed between print media categories

Attempting to distill the themes of each written media portions to a single topic, the academic press prior to the 2013 outbreak focused on the virus. The academic press after the onset of the outbreak instead focused on the disease. Magazines instead focused on the individual providers, often interviewing them directly for quoted insights. Newspapers focused on containment, both the measures to limit spread within and between nations. Blog posts tended to focus on the toll, outlining the cases and fatality. When accurately discussing the outbreak, alternative sites focused on geopolitical topics such as the choice for President Obama’s Ebola Czar.

#### Errors were of limited types

A complete list of errors is provided (S2 Table). Many sources mentioned bleeding without detailing that this is not a universal finding. Only roughly half of patients with EVD develop signs of bleeding [1]. While bleeding is associated with risk for negative outcomes, the lack of universality is an important caveat for diagnosing clinicians. As stated in the methods, articles were scored independent from the known literature to assess the outcome if a reader took the words as actionable intelligence. Since numerous diseases in Western Africa can mimic EVD symptoms [1], any clinician that mistakenly believed bleeding was a central finding in EVD would risk misdiagnosis. Therefore, statements of anemia or bleeding without mention that clinicians should not relay on such findings, especially in the early stages of the disease, were documented as erroneous due to being potentially dangerously incomplete.

Numerous academic journal articles provided errors of omission for PPE. Given that the official CDC recommendations on PPE shifted during the outbreak thereby creating a moving target on accuracy of PPE statements, PPE incompleteness was scored against the original and least complex recommendations. Statements included “Patients must be isolated in a manner that prevents exposure to their blood and body fluid (i.e., droplet/contact precautions) with health care workers using the appropriate personal protective equipment (fluid-impermeable gowns, gloves, respiratory protection, and eye protection)” [9]. An error of omission was scored if the author(s) did not make it clear that their list of PPE supplies was not all-inclusive. Reader supplied with gloves, gowns, and a facemask that presumed that the PPE list was complete would risk serious consequences. In contrast, authors stating that PPE “includes” certain items were not scored as erroneous as such language implies incomplete itemization. This is akin to writing a cake recipe and listing needed supplies as ‘an oven, flour, and sugar’ versus ‘includes an oven, flour, and sugar’.

Alternative media errors were commonly related to an accusation that the outbreak was a hoax orchestrated by politicians as an excuse to institute marshal law. While no other media type shared these errors, the alternative site’s anti-government sentiment around either an over-reaction or inadequate response was shared by many of the newspaper op-ed articles.

The most frequent error across all media was confusion over the airborne transmission potential for EVD. Two studies cited by the review articles selected raise the suggestion of airborne transmission; the first included intentionally spraying primates with a blood-containing aerosol contaminated with Ebola virus. Effectively the researchers placed the heads of the study primates under a fish tank and pumped in a fog of Ebola virus [121]. The purpose of this study was to assess the potential for Ebola virus to be employed as a weapon of biologic warfare. The findings indicate that EVD could be spread by an intentional release within an area of poor ventilation such as a subway car or plane. However, these findings do not indicate that EVD is ‘airborne’ under natural conditions. In fact, similar studies have been done with Hepatitis B virus showing that a blood-containing aerosol created if a high-powered dental drill contacts the gums could create an infectious inhalation [122, 123].

Another study suggested that pigs infected with EVD might transmit infection to primates [124]. Pigs infected with Ebola virus were housed above non-human primates and the primates were monitored for the onset of the disease. While the primates did contract EVD, the study was designed only to see if spread from pigs to primates was possible, and the researchers own commentary stated that they could not distinguish if the spread was by aerosols or droplets. While airborne spread could not be excluded, the authors themselves stressed that the far more likely mechanism of transmission was from the bloody secretions from the pigs falling into the primate enclosures beneath them [124]. This misinformation took hold despite several noteworthy studies refuting the natural transmission from primate to primate by any means other than direct contact with body fluids.

The official consensus on fomite spread of EVD is that the science is unclear. There have not been reported cases connected to fomite spread. However, Ebola virions can survive on surfaces for several days to weeks and thus spread is theoretically possible [125]. However, evaluation of clinical environments showed that no viable Ebola virus could be found on any surfaces that were not visually contaminated with blood [126], indicating that EVD transmission through routine daily exposures does not occur. Evidence against natural airborne spread is even more extensive. There have been no cases of EVD suspected of aerosolized transmission. Individuals cohabitating with EVD patients, even in tight quarters, do not contract the disease without contacting blood or body fluid [1, 18, 23, 45, 78, 83, 92]. Furthermore, the R_0_ (R-naught) number for EVD is markedly below that of influenza or other diseases known to have an aerosolized potential for spread [1]. Therefore, the only correct way to discuss airborne spread for EVD is to definitively state that such spread does not occur under natural conditions. Statements of airborne transmission within a context of biological warfare were not present in the literature reviewed, but would not have been scored as erroneous. Statements made within a context of a human outbreak that suggest airborne spread ‘cannot be excluded’ or ‘may be possible’ are not consistent with the factual conclusions from the totality of the literature.

### Quantitative results

#### There were no significant associations between completeness and generalized measurements of article prestige

**Fig 2:**
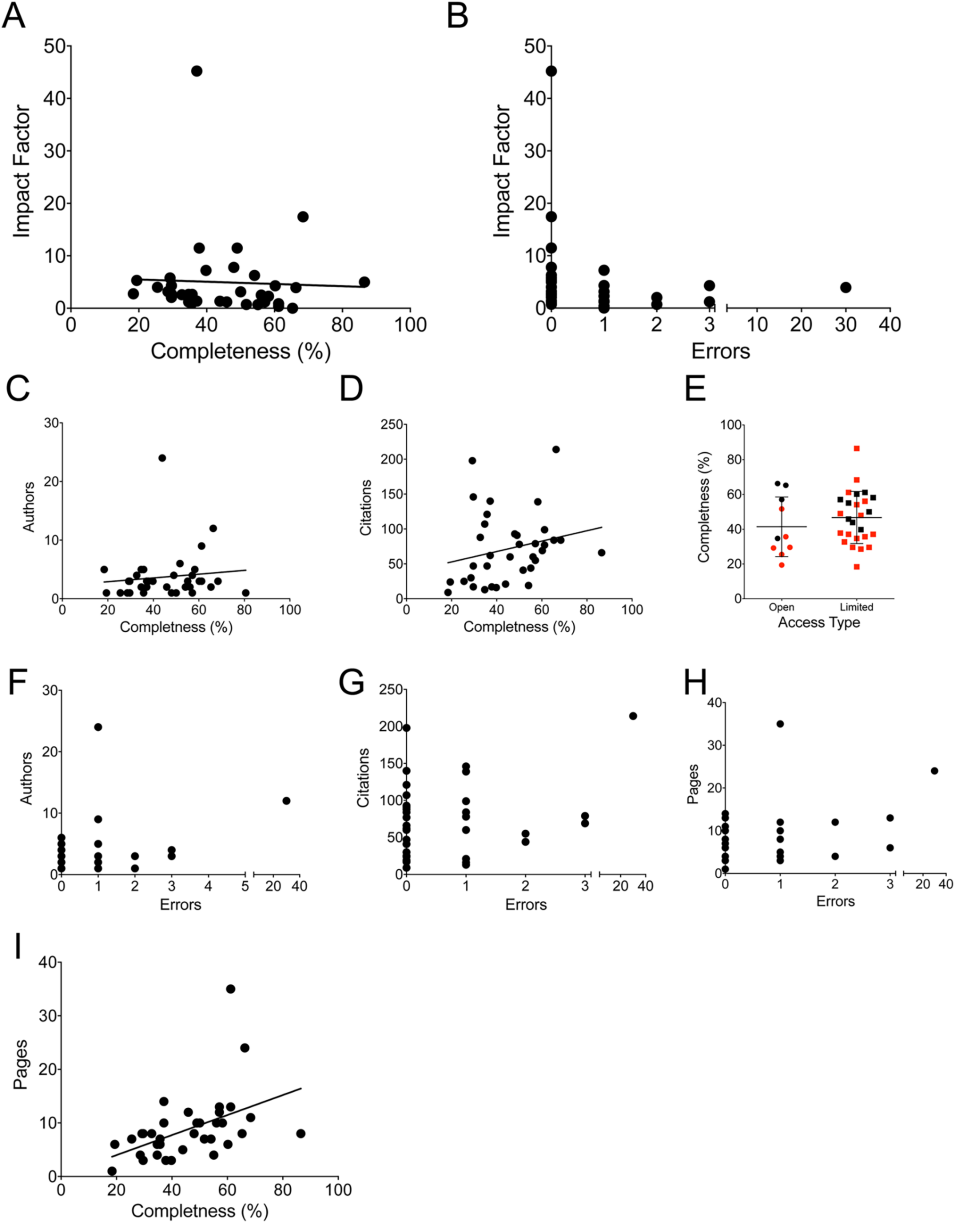
Completeness and errors did not correlate to markers of journal quality. Each academic press article is graphed for completeness versus impact factor (A), errors versus impact factor (B), completeness versus authors (C) and citations (D). (E) Completeness for full open access journals versus limited access is shown, red dots indicate articles that were free of errors. Errors versus authors (F), citations (G), and pages (H) are shown. (I) completeness versus page length is shown.

Impact factor is often considered to be the central measure of a journal’s prestige and, by proxy, the quality of the articles contained within them. However, there was no significant association between impact factor with either completeness (Fig 2A) or error rate; the articles with impact factors greater than 10 were all error free, however this did not reach statistical significance (Fig 2B). Similarly, the numbers of authors, number of citations, and whether the journal was open access also had no association with either completeness or error rate (Fig 2C–2G). Only page numbers correlated with completeness (but not errors) for academic publications (Fig 2H and 2I; p = 0.003, R^2^ = 0.234). However, a low number of printed pages did not preclude complete or accurate articles. In fact, the most complete academic paper [1] (80.6%) had no errors, only one author, and needed only 8 pages (below the average of 8.6) due to use of informative tables. This suggests that stereotypical measures of an academic paper’s status would not be a valid means for readers to pre-screen review articles for EVD.

#### Academic journals had significantly more completeness and errors per article

**Fig 3:**
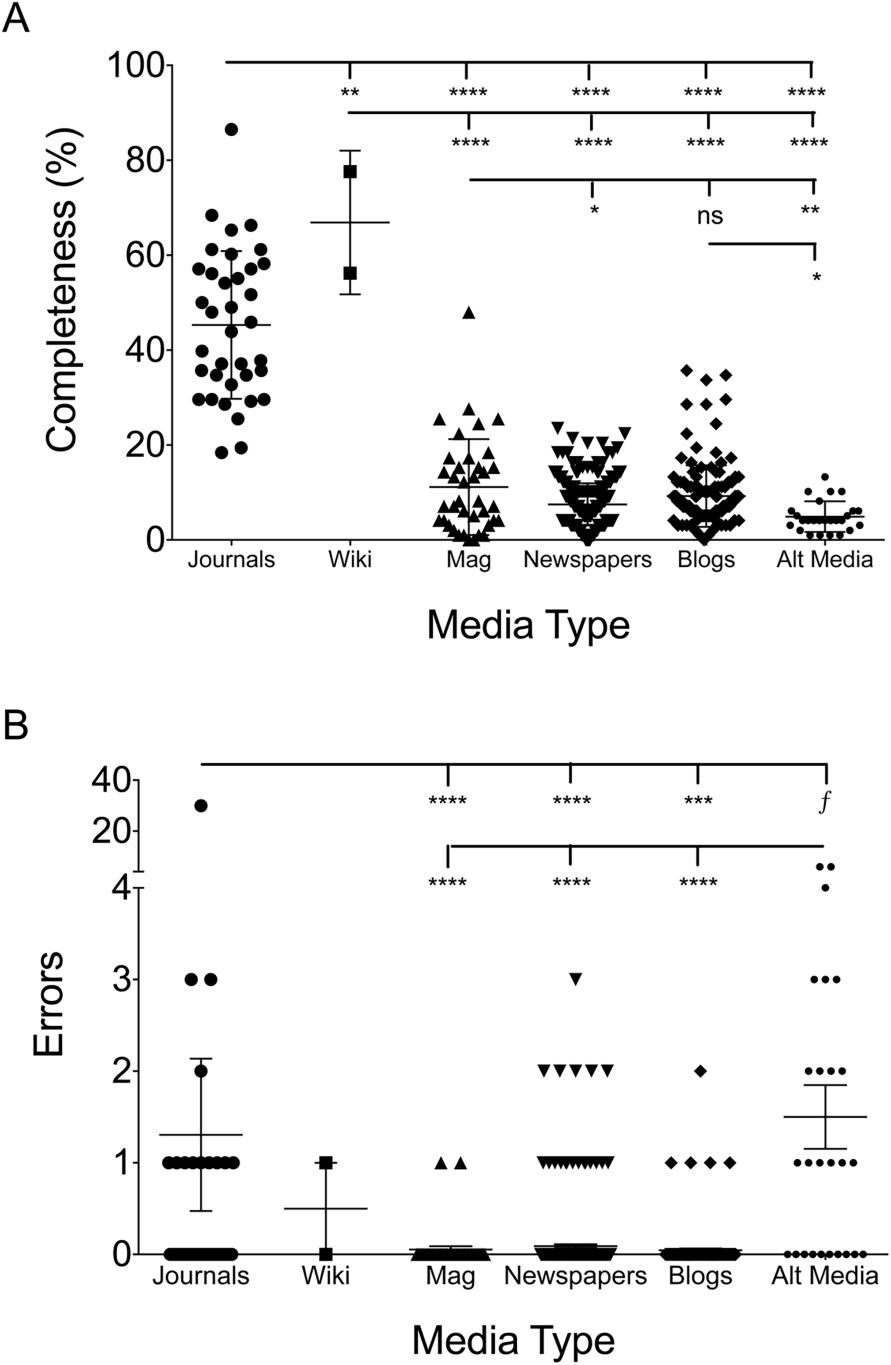
Both completeness and errors were larger in academic journals. Completeness (A) and errors (B) for each of the categories of written press. Each dot represents one article. Significance determined by ANOVA, ****—p = <0.0001, ***—p = <0.001, **—p = <0.01, *—p = <0.05, f = p value significant if outlier statistically removed from academic errors group.

As expected, academic review articles were more complete than lay press articles, as would be expected of review articles aimed at complete coverage of their designated topic. The mean academic publication covered 45.1% of the gleaned topics while magazines (11.4%), newspapers (7.1%), blogs (9.3%), and alternative sites (4.9%) lagged behind (Fig 3A). However, Wikipedia revealed one of the top scores with 77.6% after the outbreak and scored a 56.2% before the outbreak. The academic journals were typically over 8 pages while lay press articles were often less than two. Therefore, adjusted for page length there was only slightly and non-significantly higher for academic press (7% of total topics covered per page) compared to Wikipedia (5.5%), magazines (4.7%), newspapers (6.8%), blogs (6.9%), and alternative sites (4.3%). The lower score for magazines was, in part, due to greater presence of large pictures. As a somewhat unexpected finding however was that academic journals carried a higher error rate than magazines, newspapers, and blogs, but not alternative media (Fig 3B). Although there was one academic paper with 30 different erroneous statements [68], if this outlier were excluded the difference with alternative media would become significant but it would not change the significance for other lay press sources. Of note, errors in newspaper writings were associated with anonymous authorship and being a letter to the editor or editorial.

#### Completeness and errors between magazines, newspapers, blogs, and alternative sites

Magazines were significantly more complete than newspapers and alternative media (Fig 3A). No differences in accuracy were seen between magazines, newspapers, or blog posts (Fig 3B). Coupled with the indistinguishable numbers on completeness per page and errors, this may inform the debate between so called ‘new’ and ‘old’ media. Alternative media articles were significantly less complete than blogs and magazines, and contained significantly more erroneous statements than all other forms of media (Fig 3A and 3B). Of note however, there were notable differences in the focus of the coverage between lay press media (Table 3). Magazines tended to more closely mirror academic journal subject focus, covering symptoms, risk factors, pathogenesis, and virology. Blogs were more likely to cover epidemiological data, but were the least likely to discuss means to preventing spread within the population.

## Discussion

Under a traditional paradigm, only those with the expected background knowledge consume academic literature. The lay press then plays a complementary role, extracting findings of high interest or importance and translating them for general viewing. Preferably, the lay press would translate the academic range of opinion (often detailed in review articles) over what is possible into a tangible discussion on what the actionable items are for their readers. For example, rather than debate the academic nuances of a recent publication on heart disease, a newspaper may focus on communicating which, if any, life style modifications a reader should make when the new study is viewed in the context of the established review article recommendations. To some extent our findings support a potential synergistic dynamic of academia and the lay press. Had our results supported our initial hypothesis and indicated that comprehensive review articles could serve as a collective source of accurate information, the subsequent recommendations would have been to encourage lay press authors to access these journals for background understanding. However, we instead found that reviews could not reliably clarify confusion surrounding EVD nor could they combat disinformation in alternative media.

Additional interlocutors during the EVD outbreak were the web pages from agencies such as the CDC and WHO [127]. Resources and inaccessibility of contemporaneous versions of agency webpages precluded a similar assessment of these intermediaries, however these outlets can be important sources for writers in academia, media, as well as public policy. These agencies resources should receive their own evaluation as to whether they could have proved accurate contextualization of academic information for lay press background research.

The increased completeness seen in the academic review articles collected was likely a reflection of our study design rather than the authors of the individual manuscripts; general review articles were specifically targeted instead of those that may focus on only vaccine development or diagnostics and thus carried a selection offset in favor of completeness. Therefore comparing lay press articles on the same scale of our selected reviews is somewhat artificial. However, this comparison may still provide useful information by enumerating what portion of the total body of knowledge lay press article present.

The review article format is structured to present findings within the larger context, and is thus less likely than primary research to spark debate or be presented out of context. However, while the internet has greatly expanded the knowledge base within the proverbial ivory tower, it has also opened the tower to the pressures of the modern media environment in ways that can threaten the contextual foundation that review articles are expected to provide. The longstanding pressure in academia to ‘publish or perish’ has been heightened by online journals that print anything for a fee but cannot be distinguished from respected peer-reviewed counterparts by the untrained eye [128, 129]. Similarly, the longstanding lay press strain to produce scoops on a deadline has been magnified by numerous online sources participating in a minute-to-minute news cycle.

Well-meaning reporters that looked to academia for clarity would seem more likely to mistakenly concluding that airborne transmissibility was a possibility worthy of prompting policy changes if traditional measures of journal status like impact factor were of poor guidance, 1 in 3 academic papers failed to discuss airborne spread, and 1 in 3 of those that did cover it failed to communicated that no specific actions should stem from this negligible risk. As another example, a debate over the potential for EVD to be contracted from a bowling ball may never be settled in academia. Even with a dedicated study looking at bowling balls as EVD fomites, no researcher could test for all possible variables such as ambient temperature, porousness of the bowling ball, length on contact, skin integrity of the bowler, starting inoculum, etc. Communications to the public should stress that any actionable policy trying to curtail airborne EVD would waste resources and that no one should cancel their bowling plans. While the CDC may be tasked with the duty of communicating these nuances, academia in the internet-era may not be well served by the finding that many EVD comprehensive review articles were ripe for misinterpretation.

While we cannot assess causation, our results suggest that the ambiguity of academic papers created a potential for inadvertently feeding into misinformation, even if the academic literature was more reflective of the deference owed to scientific range or opinion. Speculation into the possible consequences of the mutation rate of Ebola virus [130] and the aforementioned pig-to-primate study was misrepresented as a cause for alarm on future airborne spread. Confusing word choice on when a person with EVD is contagious was misrepresented as justification for travel bans and prolonged quarantines for asymptomatic health care providers returning from overseas. Alternative sites (InfoWars 5, Beforeitsnews 18 and 26) linked directly to primary literature discussing the impact of traveler screening on mathematical models of spread [131, 132], contaminated saliva in EVD patients [133], and aerosols following toilet flushes [134] as support for their claims of the need for travel bans and proof of airborne transmission. These alternative citations were making erroneous extrapolations without misrepresenting the objective findings of each scientific paper.

While little can be done to prevent intentional misrepresentation by any media outlet, these examples prompt a question as to whether academicians need to take a more active role in communicating the actionable items in their findings directly within their manuscript, especially when the review format allows for synthesizing published information into a complete narrative. The simplest and perhaps most ideal approach that academicians writing about topics of public interest may consider is to summarize the current approach to the problem they are addressing. Word limits in publications may make it difficult to spend any space on what should be considered background knowledge of the reader, however scientists could also consider using social media and press releases to directly communicate the actionable items in their findings to serve as both scientific speaker and lay translator [135].

Another approach may be, when possible and appropriate, to have academicians use Bayesian prediction modeling to calculate odds on their statements of ‘possibility’ as a means of allowing for a more serious presentation regarding the need to act when translated for the public. No pathogen in the history of known biology has even mutated to change its mechanism of transmission [136, 137]; therefore there are more publications supporting the position that the universe is a hologram [138–140] than blood borne viruses spontaneously becoming airborne transmittable. Such modeling would be difficult, but could include the average viral load of EVD patients (roughly 10 million) [141] multiplied by the total documented cases in history (31,079) [142] to get an estimate of the total interactions between an Ebola virion and the human host. This may support the estimate that historically, roughly 300 billion virion-host interactions have occurred without any reported airborne transmission. It is not our intention to proffer this 1 in 300 billion estimate as the definitive pre-test odds calculation, and any post-test assertions would have to account for viral turnover, predictions of undocumented cases, animal carriage, and other factors. While a more Bayesian approach [143, 144] would itself generate debate within the scientific community, a reporter attempting to make a last minute deadline would find more than just the terms “possible” or “cannot be excluded” as stand ins for every probability between impossible and almost certain. This may better communicate whether the current EVD control measures should be modified to address the chance of airborne spread by enumerating the remoteness of the risk for their readers.

For those scientific problems for which odds could not be postulated without undesirable subjectivity, perhaps illustrative comparisons could be made; as one hypothetical example, comparing the risk of airborne Ebola virus to that of airborne HIV. An additional option may be to co-opt terms from the intelligence community wherein ‘probably not’, ‘probably’, and ‘almost certainty’ denote distinct odds calculations [145] just as the terms ‘trend’ and ‘significant’ have been used to denote mathematical value. Additionally, academic authors could look to recent evidence on clearer public communication through use of a vernacular that the public is more likely to understand [146]. For example, use of the word ‘enhance’ by scientists was meant to convey ‘intensify’ but was perceived by the public to mean ‘improve’ [146].

Even if concern over the potential perils of presenting odds in the peer-review process are insurmountable, academic writers might aide in collective understanding by specifying if changes in public policy are warranted rather than defaulting to a potentially misleading ‘possible versus impossible’ dichotomy. This approach could make it easier for media outlets to fact-check the claims of competing sources that conflate possibility with likelihood either unintentionally or for purposes of fear mongering.

While low scientific literacy [147] and over-simplified media coverage [148] bare some responsibility for the public confusion, researchers must ask themselves what degree of the public’s failure to glean actionable items from their studies is a reflection on academia itself. At minimum, academicians must be mindful of the context within which they present scientific uncertainty. Data related to aerosolization of Ebola virus within a context of bio-warfare preparation present a different risk of misinterpretation when the context of the discussion shifts to that of outbreak response; efforts must therefore be made to present the actionable implications of the data within the new context. This clear communication is especially vital during the height of an epidemic when state-enforced measures can worsen local social insecurity [120] whilst the truly “viral” spread of public attention creates a susceptibility to a fear contagion [149].

When viewed qualitatively, alternative media outlets actively presented disinformation. However, other lay press sources not only made accurate statements regarding the body fluid transmission of EVD, many flatly stated “Ebola is not an airborne disease”. This could indicate that public health officials and academicians may find a willing partner in some written press sources; those from which most Americans get their news appear to aide in fostering an accurate understanding of serious medical topics. These finding may also represent a potentially useful addition to news sourcing algorithms, such as those used by Facebook [150], to preference media outlets with objective track records of accuracy and completeness. Interestingly, there were no significant differences between new and old media for the most popular news outlets. It would be intriguing to hypothesize if popularity of these sources is a reflection of their apparent dedication to accuracy that begets selective advantage in the marketplace, or if their built-brands afford them enough cover to avoid stereotypical ‘click-bait’ or sordid sensationalism.

However, the lay press may require a re-evaluation of the approach to editorials and letters to the editor. Of the 27 errors made in the newspaper media, 19 (70%) were within the categories of letters or editorials. Furthermore, the quality of the errors within the letters and editorials were also far more misleading. While the standard news pieces mostly erred in not specifically stating that bleeding is not a universal symptom of EVD, the letters and editorials misled by stating travel bans would be a net benefit, airborne spread is an actionable concern, and patients were contagious before being symptomatic (S2 Table). While the alternative media articles used more apocalyptic verbiage, the basic themes of their errors did not differ from newspaper op-ed and letters. While it is understandable that newspapers would want to solicit input from a diverse sampling of sources, perhaps they should hold these authors the same standard of factuality that they hold their staff writers to, or consider editor annotation to point out misinformed or misleading statements.

The major limitation in this study is that the lay press collected was not all-inclusive. It was not feasible with the resources at hand to collect all media coverage, including television and less popular magazines, newspapers, and blogs. Even within the media outlets selected, many of the lay press outlets do not have an objective means of collecting all relevant publication; as such, only a sampling of lay press articles could be included in this analysis. However, the ease of accessibility of the media included did not predict the content or accuracy; newspaper articles covered in the *New York Times* did not differ significantly from the completely accessible *Wall Street Journal* except that the later contained editorials that carried an increased risk of erroneous statements. Furthermore, this study’s use of objective mechanisms of collecting the lay press sources excluded some that are specialized for scientific information (such as *Science News* or *Scientific American*). An interesting follow up study could be to assess if these science-focused outlets were equal or superior to the outlets collected in this investigation. While this would not be conclusive, it does suggest that the large sample examined, while incomplete, was reflective of the whole. An additional major limitation is the inability to combine both qualitative and quantitate analysis in an objective manner. The binary scoring of the CERC system meant that articles that simply mentioned “community outreach” were given an equal score as those that detailed the types and benefits of such practices. It could be valuable to perform subsequent analysis re-scoring papers based on depth of each major topic, if this could be done in an objective fashion.

Another limitation is that the focus on EVD may not allow readers to extrapolate to other issues of health or medicine. Furthermore, we cannot draw causation from our findings. It is unknown if any reporters, politicians, or pundits made inaccurate statements due directly to the lack of clarity in the academic reviews. However, this work may still achieve its goal of comparing the contemporaneous academic reviews available to reporters and policy makers at the time of their background research. Lastly, this analysis stops with the outbreak’s containment, which therefore overlooks the long-term chronic pains and visual changes that have plagued some EVD survivors [151].

President Taft is credited with advising, “don’t write so that you can be understood, write so that you can’t be misunderstood”. While this study in no suggests that the lay press was superior to academic works, the crux of our conclusions is that modern media access to academia makes it far more likely that a given academic author will be misunderstood. Writing within academia affords the ability to focus on novel findings and forego discussion of background knowledge that the author can safely assume the reader possesses. However, seclusion from the populace also carries a disadvantage of needing to rely upon the lay press to translate one’s findings into that which is fit for consumption in the public sphere. Given the erosion of the barriers siloing an academia increasingly pressured to produce publications, combined with the demands of today’s fast-paced media environment, contemporary researchers should realize that no study is outside the public forum and to therefore consider shifting the traditional paradigm to take personal responsibility in the process of accurately translating their scientific words into public policy actions.

## Supporting information

S1 Table Full results from scoring. All collected and scored documents are listed for content of all CERC categories. 1 = topic was covered, 0 = topic was not covered. Calculations for averages among sources and genres are also included.
(XLSX)

S2 Table Error list. Listing and description of all errors scored across media outlets and genres.
(XLSX)

S1 Appendix Complete list of lay press citations. Full listing of all lay press articles collected and scored. Number corresponds to text references and S1 Table listings.
(DOCX)
