# Supplementary material for: Contrasting academic and lay press print coverage of the 2013-2016 Ebola Virus Disease outbreak
Source: PLoS One. 2017 Jun 22;12(6):e0179356. doi: 10.1371/journal.pone.0179356 (PMC5480889; doi:10.1371/journal.pone.0179356)
Supplement: S1 Appendix — Full listing of all lay press articles collected and scored. Number corresponds to text references and S1 Table listings. (DOCX) [file pone.0179356.s003.docx]

**Supplemental Appendix 1: Lay Press Citations.**

***Wikipedia:***

1. Ebola Virus Disease. Accessed June 14, 2016 from <https://en.wikipedia.org/wiki/Ebola_virus_disease>. Prior version (Dec 9, 2013) accessed June 14, 2016 from: <https://en.wikipedia.org/w/index.php?title=Ebola_virus_disease&oldid=695777031>

**Magazines:**

*AARP Magazine*

1. Goodwin, Jan. Back From the Dead: An Ebola Victim’s Story. Dec2014-Jan2015

*Cosmopolitan*

1. Peikoff, Kira. 10 Things More Likely to Kill You Than Ebola. Oct 22, 2014.
2. Barker, Emma. Ebola Could Become An STD. Oct 31, 2014.
3. Rees, Alex. Ebola Nurse Nina Pham Will Sure Her Hospital. March 2, 2015.
4. Koman, Tess. Hostpial Releases Emotional Video of 26-Year-Old Ebola Nurse. Oct 17, 2014.
5. Moore, Lane. How Authorities Ruined This Ebola Survivor’s Wedding. Nov 7, 2014.
6. Thompson, Eliza. Jennifer Lawrence Stars in Somber PSA About Ebola. Dec 30, 2014.
7. Harris, Jamie. NBC’s Dr. Nancy Snyderman Breached Her Ebola Quarantine to Go Get Some Lunch. Oct 14, 2014.
8. Rees, Alex. Ohio Wedding Boutique Closed Following Reports That Ebola-Stricken Nurse May Have Been Symptomatic When Trying on Gowns. Oct 17, 2014.
9. Thompson, Eliza. One Direction, Rita Ora, and a Whole Bunch of Other People Team Up to Fight Ebola. Nov 17, 2014.
10. Friedman, Megan. One of the Ebola Nurses Is Getting a Free Engagement Ring. Nov 20, 2014.
11. Beck, Laura. The Sexy Ebola Costume Is Here. Oct 26, 2014.
12. Narins, Elizabeth. This Fashion Blogger in Sierra Leone is Now Documenting the Ebola Outbreak. Oct 2, 2014.
13. Narins, Elizabeth. What It’s Like to Survive Ebola. Oct 25, 2014.
14. Narins, Elizabeth. What It’s Like to Be an Aid Worker in Sierra Leone During the Ebola Crisis. Oct 3, 2014.

*Glamour Magazine*

1. Petronis, Lexi. Comforting Facts to Know If You’re Freaking Out About Ebola. Oct 13, 2014.
2. Eidell, Lynsey. So, It Looks Like an Ebola Television Show Is in the Works. Oct 17, 2014.
3. Petronis, Lexi. Ebola Has Reached the US, but Here’s Why You Shouldn’t Freak Out. Oct 1, 2014.

*National Geographic*

1. Quammen, David. Seeking the Source of Ebola. July 2015.

*TIME Magazine*

1. West Africa’s Ebola Outbreak. Explainer. Aug 18, 2014.
2. Walsh, Bryan and A. Sifferlin. After Ebola. Aug 25, 2014.
3. Von Drehle, David. Now Arriving. Oct 13, 2014.
4. Von Drehle, David. The New Ebola Protocols. Oct 27, 2014.
5. Walt, Vivienne. Missing In Action. Nov 10, 2014.
6. Gibbs, Nancy. The Choice. Dec 22, 2014
7. Kluger, Jeffery. Fear Factor. Oct 20, 2014.
8. Baker, Ayrn. Liberia Struggles Against Ebola. Dec 29, 2014.
9. Sifferlin, Alexandra. Recovered: Dr Kent Brantly and Nancy Whitebol. Sept 8-15, 2014.
10. Briefing. Lets Hug For the Cameras. Nov 10, 2014.
11. Dias, Elizabeth and N Rayman. The Worst Ebola Outbreak on Record. Dec 1-8, 2015.
12. Milestones. Died. Dec 1-8, 2015.
13. Sifferlin, Alexandra. A Year of Ebola. April 6, 2015.
14. Von Drehle, David. The Ones Who Answered The Call. Dec 22, 2014.
15. Fischer, William. Ebola Doctors. Aug 18, 2014.
16. Redd, John. Biological Warfare. Dec 1-8, 2014.
17. Park, Alice. Recovered: Craig Spencer. Nov 24, 2014.
18. Gibbs, Nancy. A Risky Business. Oct 20, 2014.
19. Altman, Alex; A. Park, T. Berenson, Z. Miller, J. Sanburn, A. Sifferlin, and B. Saporito. 12 Answers to Ebola’s Hard Questions. Nov 3, 2014.

**Newspapers:**

*Los Angeles Times*

1. Zavis, Alexandra. The Ebola Flare-up risk; Staying vigilant is key to fighting virus, which requires research. Jan 15, 2016. A2.
2. Morin, Monte. State is Ending Ebola Checks. Jan 8, 2016. B4.

*USA Today*

1. Weintraub, Karen. Drug found effective against virus similar to Ebola; New approach months from helping very many. Aug 21, 2014. 3A.
2. Szabo, Liz. West Africa’s Ebola survivors face lingering health issues; Many are unable to work because of joint pain, eye trouble or trauma. Aug 10, 2015. 5A.
3. Szabo, Liz. Calm Down, you won’t catch Ebola. Oct 3, 2014. 2A.
4. Szabo, Liz. Ebola demands humanity, not hysteria. Oct 17, 2014. 2A.
5. Szabo, Liz. Not Sending Doctors is Bigger Risk Than Ebola. Oct 27, 2014. 2A
6. Bialik, Carl; W.F.B. O;Reilly, L. Garrett, Christian Science Monitor editorial board, K.Y. Dionne. Ebola outbreak a cause for alarm of for calm? Aug 4, 2014. 8A.

*Washington Post*

1. Kamen, Al and C. Itkowitz. We’ve heard from the doctors. Here come the lawyers! Oct 30, 2014. A15
2. Rettner, Rachael. Attention shifts to the long-term effects of Ebola. March 10, 2015. E04

*New York Post*

1. Perez, Chris and M. Gartland. Ebola kin lawyer up. Oct 21, 2014. 17.

*The New York Times*

1. Barash, David and L. Black. Keeping Ebola at Bay. Dec 1, 2015. Letter.
2. Dhillon, Ranu; D. Srikrishna and R.F. Garry. Testing for Ebola. Feb 10, 2016. Letter.
3. McNeil, Donald. Ask Well. Nov 4, 2014. D6.
4. McNeil, Donald. Can You Get Ebola From a Bowling Ball? Oct 28, 2014. D4.
5. Olsen, Patricia. On the Lookout for Ebola. Oct 19, 2014. B7.
6. McNeil, Donald. How Does Ebola Spread? How Long Can the Virus Survive? Oct 7, 2014. D4.
7. Fink, Sheri. Indefinite Safe Sex Urged for Liberia Ebola Survivors. March 29, 2015. A12.
8. Reuters. Ebola Patient Discharged, Last of the Country’s Latest Wave. July 21, 2015. A8.
9. Searcey, Dionne and S. Fink. Day After a Victory Over Ebola, Sierra Leone Reports a Death. Jan 15, 2016. A4.
10. Belluck, Pam. Emergency Over Ebola Has Ended, WHO Says. March 29, 2016. A6.
11. Cumming-Bruce, Nick. Nigeria is Free of Ebola, Health Agency Affirms. Oct 21, 2014. A16.
12. Searcey, Dionne and S. Fink. Sierra Leone Declared Free of Ebola Transmissions. Nov 8, 2015. A8.
13. The NYT Editorial Board. The Ebola Outbreak. July 30, 2014. Editorials.
14. Searcey, Dionne. The Last Place on Earth With Ebola: Getting Guinea to Zero. Nov 7, 2015. A1.
15. Dahn, Bernice; V. Mussah and C. Nutt. Yes, We Were Warned About Ebola. April 8, 2012. Editorials.

*New York Daily News*

1. Engle, Meredith. Name-calling is sick: nurse. Nov 18, 2014. News 16.
2. Hamill, Denis. Panicking is far more contagious. Oct 25, 2014. News 6.
3. Hutchinson, Bill. Ebola nurse;s pooch is fine. Oct 23, 2014. News 19.
4. Hamill, Denis. Ebola is hardly our top worry scare this H’ween. Oct 16, 2014. News 6.
5. Anonymous. How it got loose in US. Oct 16, 2014. News 4.
6. Hutchinson, Bill. Quick facts about the deadly outbreak. Oct 24, 2016. News 5.

*The Wall Street Journal*

1. Hinshaw, Drew. World News: Dozens Die As Ebola Outbreak Hits Guinea. Mar 25, 2014. A7.
2. WSJ Roundup. Liberian doctor dies from Ebola. Jul 28, 2014. A8.
3. Hinshaw, Drew and B. McKay. West Africa strains to contain Ebola virus – Liberia closes most borders, bans large gatherings as toll mounts; CDC issues health alert, but calls risk to US low. Jul 29, 2014. A5.
4. Associated Press. Doctor leading fight against Ebola in Sierra Leone dies after contracting virus. Jul 30, 2013. A7.
5. McKay, Betsy. Aid workers flee Ebola threat. Jul 31, 2014. A14.
6. Hinshaw, Drew and B. McKay. Ebola outbreak sparks quarantine. Aug 1, 2014. A7.
7. McKay, Betsy and C. McWhirter. Two patients bound for US. Aug 2, 2014. A1.
8. Hinshaw, Drew and B. McKay. Africa's Largest City Resists Ebola Threat. Aug 2, 2014. A5.
9. McKay, Betsy and C. McWhirter. Isolated Ebola patient faces crucial days. Aug 4, 2014. A3.
10. Lipkin, W Ian. Ebola: how worried should we be? Aug 4, 2014. A13.
11. Hinshaw, Drew. Ebola mars Africa summit aimed at luring US firms. Aug 4, 2014. A14.
12. Loftus, Peter and C. McWhirter. Patients got unapproved Ebola drug. Aug 5, 2014. A3.
13. Hunstberry, Will. Mount Sinai treats patient with Ebola-like symptoms. Aug 5, 2014. A17.
14. McWhirter, Cameron, P. Loftus and D. Hinshaw. Giving Americans Ebola drug prompts flak. Aug 6, 2014. A3.
15. Hunstberry, Will. Hospitals laid plans for Ebola. Aug 6, 2014. A15.
16. Farrar, Jeremy, D. Heymann and P. Piot. Experimental medicine in a time of Ebola. Aug 7, 2014.
17. Hinshaw, Drew and G. Akingbule. African health workers feel Ebola’s wrath. Aug 7, 2014. A12.
18. Hinshaw, Drew and L. Radnofsky. Two African nations impose Ebola quarantines. Aug 8, 2014. A8.
19. Hinshaw, Drew, S. Wang and J. Miller. Ebola overwhelming aide effort. Aug 9, 2014. A5.
20. Konneh, Amara. The Economics of Ebola. Aug 11, 2014. A13.
21. Hinshaw, Drew and P. McGroaty. African nations curb flights in Ebola fight. Aug 12, 2014. A6.
22. McKay, Betsy and P. Loftus. Experimental drugs are approved to fight Ebola in West Africa. Aug 13, 2014. A7.
23. McKay, Betsy. Fear of Ebola spread prompts extraordinary precautions. Aug 14, 2014. A3.
24. Armental, Maria. Tekmira assesses experimental Ebola drug. Aug 14, 2014. B4.
25. Akingbule, Gbenga and H. Vogt. Nigeria to try new drug on Ebola victims there. Aug 15, 2015. A7.
26. Hinshaw, Drew. On the front lines: for want of gloves, Ebola doctors die. Aug 16, 2014. A1.
27. McKay, Betsy. Doctor hopes to leave Ebola ward soon. Aug 16, 2014. A3.
28. McKay, Betsy. Second US Ebola patient also improving. Aug 19, 2014. A6.
29. Vogt, Heidi. Ebola taking toll on African tourism. Aug 20, 2014. A8.
30. Hinshaw, Drew. Army fires at Liberians fleeing quarantine. Aug 21, 2014. A14.
31. McWhirter, Cameron and B. McKay. American Ebola patients released. Aug 22, 2014. A3.
32. McKay, Betsy, N. Bariyo and D. Hinshaw. A tale of two Africa’s. Aug 23, 2014. C1.
33. Hinshaw, Drew and B. McKay. New Ebola outbreak shows larger threat. Aug 26, 2014. A9.
34. Miller, Henry. Genetic engineering and the fight against Ebola. Aug 26, 2014. A13.
35. Wall, Robert and B. McKay. Airline cuts complicate efforts in fighting Ebola. Aug 28, 2014. A7.
36. Hinshaw, Drew and B. McKay. Ebola worsens for lack of global aid. Aug 29, 2014. A7.
37. Hinshaw, Drew. Makeshift burials hinder battle on Ebola. Sep 2, 2014. A9.
38. Gottlieb, Scott and T. Troy. Heading off a bigger Ebola catastrophe. Sep 2, 2014. A13.
39. Audi, Tamara, C. McWhirter and B. McKay. Ebola strikes third missionary. Sep 9, 2014. A3.
40. McKay, Betsy and J. Kamp. Physician with Ebola understanding the risks. Sep 4, 2014. A6.
41. Stevis, Matina and N. Bariyo. Ebola toll on Africa economy grows. Sep 5, 2014. A9.
42. Foreman, Amanda. Historically speaking: From Typhoid Mary to the Ebola outbreak. Sep 6, 2014. C12.
43. Hinshaw, Drew and B. McKay. Deadly disappointment awaits at Ebola clinics. Sep 8, 2014. A1.
44. Stevis, Matina. Ebola-tied bans on travel to ease. Sep 9, 2014. A13.
45. Hinshaw, Drew. Ebola workers fill gaps in care by lifting spirits. Sep 13, 2014. A7.
46. Lee, Carol and B. McKay. Obama plans major Ebola offensive. Sep 15, 2014. A6.
47. McKay, Betsy. Military to send 3,000 to battle Ebola virus. Sep 16, 2014. A5.
48. McKay, Betsy and J. Forsyth. Ebola pledges lagged as epidemic widened. Sep 16, 2014. A7.
49. McKay, Betsy and D. Nissenbaum. Ebola is new foe for military. Sep 17, 2014. A3.
50. Gottlieb, Scott and T. Troy. Ebola’s warning for an unprepared America. Sep 17, 2014. A15
51. Barnes, Julian. Army chief calls for rethink of cuts. Sep 20, 2014. A4.
52. Wexler, Alexandra and S. Jerving. Cocoa surges on Ebola concerns. Sep 20, 2014. B1.
53. Loftus, Peter. New Ebola drug gets nod from FDA. Sep 23, 2014. B4.
54. McKay, Betsy. Health agencies raise Ebola alarm. Sep 24, 2014. A11.
55. Lee, Carol and J. Sparshott. Obama slams Ebola delays. Sep 26, 2014. A9.
56. Hinshaw, Drew and B. McKay. US troops battling Ebola get off to slow start. Sep 29, 2014. A8.
57. McKay, Betsy and A. Campoy. Ebola diagnosed in Texas patient; first US case. Oct 1, 2014. A1.
58. Koppel, Nathan, D. Frosch and B. McKay. Ebola patient’s contacts pursued. Oct 2, 2014. A1.
59. Campoy, Ana, D. Hinshaw and D. Forsch. US Ebola screening grows. Oct 3, 2013. A1.
60. Levitz, Jennifer. American journalist test positive for Ebola in Liberia. Oct 3, 2014. A6.
61. McKay, Betsy and D. Hinshaw. Lack of qualified staff in Africa hurts effort. Oct 3, 2014. A6.
62. Carey, Susan. United air contacting passengers on two planes. Oct 3, 2014. A6.
63. Campoy, Ana, N. McCain and N. Koppel. US tries to calm public on Ebola. Oct 4, 2014. A1.
64. Hinshaw, Drew, L. Alpert and J. Levitz. American cameraman to get care in Omaha. Oct 4, 2014. A6.
65. Geiger, Friedrich. Germany flies Ugandan doctor to Frankfurt for medical treatment. Oct 4, 2014. A6.
66. Burton, Thomas. Ebola vaccine safety study could expand by early 2015 in West Africa. Oct 4, 2014. A6.
67. Anonymous. The Ebola stand. Oct 4, 2014. A12.
68. Gottlieb, Scott and T. Troy. Stopping Ebola before it turns into a pandemic. Oct 4, 2014. A13.
69. Campoy, Ana and B. McKay. Ebola case points out flaws. Oct 6, 2014. A3.
70. Wonacott, Peter. Analysis: US-Africa business vision clouds up. Oct 6, 2014. A12.
71. Hinshaw, Drew. Liberian farm stymies Ebola’s spread. Oct 7, 2014. A1.
72. Neumann, Jeannette and I. Brat. Spain case is the first caught outside Africa. Oct 7, 2014. A8.
73. Lee, Carol and J. Sparshott. US considers stepped-up screening of air passengers. Oct 7, 2014. A8.
74. Roman, David, C. Bjork and S. Wang. Spain struggles to contain Ebola concerns. Oct 8, 2014. A6.
75. West, Melanie Grayce. Doctors prep for Ebola. Oct 8, 2014. A15.
76. Bustillo, Miguel, A. Campoy and B. McKay. Ebola patient dies in Texas. Oct 9, 2014. A1.
77. Loftus, Peter. Researchers aren’t ruling out treatment used in Dallas case. Oct 9, 2014. A12.
78. Hinshaw, Drew. UN says health worker stricken. Oct 9, 2014. A12.
79. Nicas, Jack and A. Grossman. What screening means for air travels. Oct 9, 2014. A12.
80. Brat, Ilan, J. Neumann and C. Bjork. Human error cited in Spain contagion. Oct 9, 2014. A13.
81. McKay, Betsy. Health workers prepare for the front lines. Oct 9, 2014. A13.
82. Brat, Ilan and C. Bjork. Public outcry can’t save aide’s dog. Oct 9, 2014. A13.
83. Anonymous. The Ebola democrats. Oct 9, 2014. A16.
84. Hinshaw, Drew and B. McKay. Unlikely partner: Cuba stands at forefront of Ebola battle in Africa. Oct 10, 2014. A1.
85. Neumann, Jeannette. Spanish patient’s condition gets worse. Oct 10, 2014. A10.
86. Associated Press. More Marines land in hot zone as West African leaders plead for international help. Oct 10, 2014. A10.
87. Peterson, Kristina and J. Barnes. Congress holds up funding to fight virus. Oct 10, 2014. A10.
88. Gross, Jenny. OK to screen passengers as possible new cases arise. Oct 10, 2014. A11.
89. Bariyo, Nicholas and P. McGroarty. Rising food prices make Africa fight virus on empty stomach. Oct 10, 2014. A11.
90. Tangel, Andrew, J. Jackson and M. Trottman. Calls for calm as Ebola fears grow. Oct 10, 2014. A17.
91. Frosch, Dan, A. Campoy and N. Koppel. Liberian’s days in Dallas: joy, then sorrow. Oct 11, 2014. A6.
92. Mauldin, William and K. Peterson. World bank president faults response. Oct 11, 2014. A6.
93. Johnson, Reed. South America’s first case? Oct 11, 2014. A6.
94. McKay, Betsy, M. Bustillo and M. Beck. Ebola case puts focus on safeguards. Oct 13, 2014. A1.
95. Bustillo, Miguel and A. Campoy. Second patient fuels unease in jittery Dallas. Oct 13, 2014. A6.
96. Vogt, Heidi. Instead of bodies, some teams collect bribes. Oct 13, 2014. A7.
97. Vogt, Heidi. Liberian health workers plan strike. Oct 13, 2014. A7.
98. McKay, Betsy and P. Loftus. Ebola response strains hospitals. Oct 14, 2014. A1.
99. Campoy, Ana and D. Frosch. Dallas nurse with Ebola is 2010 graduate. Oct 14, 2014. A6.
100. McKay, Betsy, T. Burton and A. Campoy. US rethinks response, focuses on lapses. Oct 14, 2014. A7.
101. Norman, Laurence. Europe plans to discuss screening. Oct 14, 2014. A7.
102. Rienhard, Beth and A. Stephanie. Crisis becomes fodder for campaign season. Oct 14, 2014. A7.
103. Wolfe, Nathan. No more Ebola Whac-a-mole. Oct 14, 2014. A15.
104. Vogt, Heidi. Ebola survivors care for new victims. Oct 15, 2014. A1.
105. Armour, Stephanie. By thin margin, many think US is prepared for virus. Oct 15, 2014. A8.
106. Baurerlein, Valeria and M. Bustillo. CDC promises faster response to outbreak. Oct 15, 2014. A8.
107. Morse, Andrew. WHO says epidemic is gaining momentum. Oct 15, 2014. A9.
108. Brat, Ilan, C. Bjork and J. Neumann. Spain case shows challenge for west. Oct 15, 2014. A9.
109. Torrey, E. Fuller. How the US made the Ebola crisis worse. Oct 15, 2014. A19.
110. Saul, Michael. City news: De Blasio talks security, Ebola in Washington. Oct 15, 2014. A23.
111. Feintzeig, Rachel. When Ebola is a workplace issue. Oct 15, 2014. B7.
112. Nicas, Jack, A. Campoy, and B. McKay. New push to check spread of Ebola. Oct 16, 2014. A1.
113. Palazzolo, Joe. Travel controls rest with multiple government levels. Oct 16, 2014. A8.
114. Loftus, Peter and B. McKay. Treatment spotlight back on Atlanta. Oct 16, 2014. A8.
115. Lee, Carol and J. Sparshott. President promises review of problems. Oct 16, 2014. A8.
116. Bustillo, Miguel and D. Frosch. Dallas warns more cases possible. Oct 16, 2014. A9.
117. Schwatrz, Felicia, A. Morse, and N. Bisserbe. Dispatches. Oct 16, 2014. A9.
118. Anonymous. The Ebola twilight of public institutions. Oct 16, 2014. A18.
119. West, Melanie Grayce. Hospital, airport workers seek Ebola training. Oct 16, 2014. A24.
120. Armour, Stephanie and C. Lee. Ebola response slammed. Oct 17, 2014. A1.
121. Bustillo, Miguel and D. Frosch. Hospital denies nurses’ allegations. Oct 17, 2014. A4.
122. Dolan, Matthew and C. McCabe. Reaction to outbreak ripples across nation. Oct 17, 2014. A4.
123. Armour, Stephanie. Upbeat tone on Ebola threat questioned. Oct 17, 2014. A4.
124. Neumann, Jennifer, C. Bjork and I. Brat. Spain isolates sickened passenger. Oct 17, 2014. A5.
125. Naik, Gautam. Change to airborne spread is unlikely. Oct 17, 2014. A5.
126. Robinson, Frances, A. Thomas and J. Letzing. Dispatches. Oct 17, 2014. A5.
127. Collins, Cassandra, M. Tice and M. Mobley. Containment of Ebola and ‘First do no harm’. Oct 17, 2014. A12.
128. De Avila, Joseph. States hurry to fight Ebola. Oct 17, 2014. A15.
129. Anonymous. Delta air lines says it has seen no effect from Ebola fears. Oct 17, 2014. B5.
130. Loftus, Peter and B. McKay. Race is on for Ebola drug. Oct 18, 2014. A1.
131. McGinty, Jo Craven. The Numbers: mapping Ebola spread requires wealth of data. Oct 18, 2014. A2.
132. Winning, Nicholas, P. Vieira, D. Roman and J. Neumann. Dispatches. Oct 18, 2014. A6.
133. Beck, Melinda and B. McKay. CDC readies a revamp of its Ebola guidelines. Oct 18, 2014. A6.
134. Nissenbaum, Dion. False alarm at the Pentagon. Oct 18, 2014. A6.
135. Lazo, Alejandro, L. Stevens and M. Dolan. Texan quarantined on cruise. Oct 18, 2014. A6.
136. Lee, Carol. Democratic aide to lead response. Oct 18, 2014. A6.
137. Anonymous. Ebola political contagion. Oct 18, 2014. A12.
138. Josephs, Leslie. Trading or shipping disruptions may not be covered by insurance, contracts. Oct 18, 2014. B5.
139. Brilliant, Larry. Ebola: what should we do now? Oct 18, 2014. C2.
140. Burton, Thomas. Ebola vaccine ramps up. Oct 20, 2014. A1.
141. Barnes, Julian. Hospital guidelines to stiffen. Oct 20, 2014. A6.
142. McCabe, Caitlin. Uneasy Dallas awaits end of 21-day incubation period. Oct 20, 2014. A7.
143. McGroaty, Patrick. Many Liberian doctors are staying away. Oct 20, 2014. A7.
144. Nicas, Jack and S. Carey. Travel ban would backfire, health-policy experts say. Oct 20, 2014. A7.
145. Gottlieb, Scott and T. Troy. Ebola isn’t a messaging problem. Oct 20, 2014. A19.
146. Naik, Gautam. Study projects spread of virus. Oct 21, 2014. A10.
147. Maloney, Jennifer, S. Calvery and D. Kravitz. Fears torment US Liberians. Oct 21, 2014. A10.
148. Anonymous. Dispatches. Oct 21, 2014. A11.
149. Hinshaw, Drew, B. McKay and G. Akingbule. Fast action wins Nigeria clean bill of health. Oct 21, 2014. A11.
150. Stephens, Bret. What Ebola experts miss. Oct 21, 2014. A13.
151. MacKellar, F. Landis and J.G. Siri. Reasons to calm down about Ebola. Oct 21, 2014. A15.
152. West, Melanie Grayce. New York accelerates Ebola training. Oct 21, 2014. A17.
153. Armour, Stephanie. Americans jittery over possible exposure. Oct 22, 2014. A6.
154. Peterson, Kristina and S. Hughes. US limits passengers from stricken nations. Oct 22, 2014. A7.
155. Anonymous. Dispatches. Oct 22, 2014. A7.
156. Wonacott, Peter. Fearful Chinese firms rethink Africa. Oct 22, 2014. A7.
157. Anonymous. A political solution for the Ebola crisis. Oct 22, 2014. A14.
158. Hook, Brian. The UN agency that bungled Ebola. Oct 22, 2014. A15.
159. West, Melanie Grayce. Thousands receive Ebola training. Oct 22, 2014. A17.
160. McGroaty, Patrick. In Liberia, orphanages make tragic returns as crisis persists. Oct 23, 2014. A6.
161. McCabe, Caitlin and C. Weaver. Hospital sees decline in patients, revenue. Oct 23, 2014. A6.
162. Campoy, Ana. US to monitor travelers from virus-stricken countries. Oct 23, 2014. A6.
163. West, Melanie Grayce. Ebola fears come and go in Newark. Oct 23, 2014. A21.
164. Dawsey, Josh, A. Tangel and B. McKay. New York physician contracts Ebola. Oct 24, 2014. A1.
165. Loftus, Peter. Study to test transfusion from survivors. Oct 24, 2014. A5.
166. Vogt, Heidi. Slum fights disease with new vigor. Oct 24, 2014. A5.
167. Armour, Stephanie. US Schools respond to fears with new plans. Oct 24, 2014. A5.
168. Vogt, Heidi. Travel delays hamper African medical effort. Oct 24, 2014. A5.
169. Anonymous. Ebola in New York. Oct 24, 2014. A12.
170. Thompson, Tommy. There’s plenty of money to fight Ebola. Oct 24, 2014. A13.
171. Mann, Ted. Ebola case puts focus on Bellevue. Oct 24, 2014. A15.
172. McKay, Betsy, A. Campoy and J. Maloney. Newest case shifts nation’s Ebola plan. Oct 25, 2014. A1.
173. Tangel, Andrew, L. Brody and D. Kravitz. Doctor was quick to volunteer overseas. Oct 25, 2014. A6.
174. Letzing, John. Timetable for vaccine trials in West Africa accelerates. Oct 25, 2014. A6.
175. Taylor, Caroline and D. Wolf. Ebola travel ban won’t work with out porous borders. Oct 25, 2014. A12.
176. Jenkins, Holman. The Ebola Anti-Hysteria Hysteria. Oct 25, 2014. A13.
177. Maloney, Jennifer, S. Hollander and J. Adam. New Yorkers cope with the arrival of Ebola. Oct 25, 2014. A15.
178. Nelson, Colleen McCain, M.G. West and B. McKay. White House pushes back on state Ebola quarantines. Oct 27, 2014. A1.
179. Wonacott, Peter. Hospital shifts contagion strategy. Oct 27, 2014. A8.
180. Vilensky, Mike. New York patient receives plasma. Oct 27, 2014. A8.
181. Dawsey, Josh, E. Orden and M. Gay. Cuomo, Christie split on Ebola. Oct 27, 2014. A23.
182. Vilensky, Mike and M.G.West. Bellevue ISU team tackles Ebola case. Oct 27, 2014. A21.
183. Mims, Christopher. In the fight against Ebola, connectivity can save lives. Oct 27, 2014. B1.
184. Schwartz, Felicia. US soldiers face tests after Africa. Oct 28, 2014. A6.
185. Orden, Erica, H. Haddon and J. Dawsey. Cuomo shifts from Christie. Oct 28, 2014. A6.
186. Geshekter, Charles and W.L. Green. The CDC knows less than it asserts about Ebola. Oct 28, 2014. A18.
187. Mann, Ted and M.G. West. Bellevue frees up beds with moves to NYU. Oct 28, 2014. A21.
188. Haddon, Heather and J. Dawsey. New Jersey hospitals in spotlight. Oct 29, 2014. A6.
189. Nelson, Colleen McCain, J. Dawsey and L. Meckler. Christie raises political stakes in Ebola debate. Oct 29, 2014. A6.
190. Jenkins, Holman. Why no Ebola travel ban? Politics. Oct 29, 2014. A17.
191. Schwartz, Felicia and D. Nissenbaum. 21-day military monitoring approved. Oct 30, 2014. A8.
192. Nelson, Colleen McCain. White House ramps up response. Oct 30, 2014. A8.
193. Levitz, Jennifer and S. Calvert. Nurse’s defiance keeps her in spotlight. Oct 30, 2014. A8.
194. Anonymous. ‘The Science’ vs. Ebola. Oct 30, 2014. A20.
195. Haddon, Heather and J. Dawsey. Ebola rules under review in New Jersey. Oct 30, 2014. A23.
196. Burton, Thomas and P. Loftus. Europe’s plan for uncontrolled Ebola trial draws fire. Oct 31, 2014. A10.
197. Wonacott, Peter. Sierra Leone ambulances fuel Ebola concerns. Oct 31, 2014. A10.
198. Moreno, Jonathan and S. Xenakis. The US military mission against Ebola. Oct 31, 2014. A13.
199. Orden, Erica. Overwhelming support for forced Ebola quarantines. Oct 31, 2014. A15.
200. Tangel, Andrew, H. Haddon and J. Dawsey. Overwhelming support for forced Ebola quarantines. Oct 31, 2014. A15.
201. McKay, Betsy. ‘I may die right here’: Ebola survivors’ painful, lonely journeys. Nov 1, 2014. A1.
202. Noonan, Peggy. Declarations: From Ellis Island to Ebola. Nov 1, 2014. A13.
203. Vieira, Paul and C. Tejada. Dispatches. Nov 1, 2014. A14.
204. Armour, Stephanie. US hospitals weight staff safety. Nov 1, 2014. A14.
205. Dawsey, Josh. N.J. details Ebola policy. Nov 1, 2014. A17.
206. Marcus, Amy. Researchers eye Ebola link to rare disease. Nov 3, 2014. A8.
207. Beck, Melinda. Hospital records adapt to flag Ebola. Nov 3, 2014. B1.
208. Hinshaw, Drew. For Ebola survivors, sex crimes added risk. Nov 4, 2014. A7
209. Loftus, Peter. Racing to create a fast Ebola test. Nov 6, 2014. B8.
210. Noudali, Robin, R. Brook and H. Nevin. Second opinions on how best to keep Ebola in its place. Nov 8, 2014. A12.
211. West, Melanie Grayce and B. McKay. Last Ebola patient in US hospital set to be released. Nov 11, 2014. A2.
212. Feith, David. Ebola and American role models. Nov 12, 2014. A13.
213. West, Melanie Grayce and J. Dawsey. Behind Ebola front lines at Bellevue. Nov 12, 2014. A17.
214. Hinshaw, Drew. Mali races to rein in virus after nurse dies. Nov 13, 2014. A7.
215. Burton, Thomas. President seeking $6.2 billion to battle deadly disease. Nov 13, 2014. A7.
216. Bustillo, Miguel. Family, hospital reach a settlement. Nov 13, 2014. A7.
217. Loftus, Peter. Antiviral drug trails to launch next month. Nov 13, 2014. A7.
218. Hinshaw, Drew and P. McGroarty. Liberia ends Ebola quarantine as new case hits Mali. Nov 14, 2014. A11.
219. Mukpo, Ashoka. I got Ebola and survived. Nov 15, 2014. C3.
220. McKay, Betsy and S. Calvert. ‘Hour-by-hour situation’ for Ebola patient in US. Nov 17, 2014. A5.
221. Gross, Michael. Ebola not EMR ‘teachable moment’. Nov 17, 2014. A12.
222. Wonacott, Peter. Villiage healers cloud Ebola fight. Nov 18, 2014. A1.
223. McKay, Betsy and S. Calvert. Doctor with virus dies in a US hospital. Nov 18, 2014. A9.
224. Gauthier-Villars, David. Coordination Frustrates African Ebola Czars. Nov 19, 2014. A7.
225. McGroarty, Patrick, D. Gauthier-Villars and A. MacDonald. Mining projects take hit from Ebola crisis. Nov 19, 2014. B1.
226. Jenny, Donald. Ebola heroes’ unheroic activities. Nov 20, 2014. A16.
227. Revill, John and A. Morse. Switzerland clinic to treat Cuban doctor for Ebola. Nov 22, 2014. A7.
228. Wonacott, Peter. US buys up Ebola gear; little left for Africa. Nov 25, 2014. A7.
229. Burton, Thomas. Vaccine for Ebola found to be safe. Nov 28, 2014. A6.
230. Villars, David and J. Whalen. Ebola crisis stretches means of Doctors Without Borders. Nov 29, 2014. A6.
231. McGroarty, Patrick. Liberia leader doubles down on Ebola battle. Dec 2, 2014. A12.
232. McKay, Betsy, D. Gauthier-Villars and P. McGroarty. Doctors try survivors’ blood to treat Ebola. Dec 6, 2014. A6.
233. Bariyo, Nicholas. Ebola-hit countries face more food woes. Dec 18, 2014. A10.
234. Armour, Stephanie. Error exposes CDC worker to Ebola. Dec 26, 2014. A2.
235. McKay, Betsy and P. Wonacott. ‘There will be another’: after slow Ebola response, world seeks to avoid repeat. Dec 30, 2014. A1.
236. Flynn, Alexis. New case of Ebola treated in Scotland. Dec 30, 2014. A5.
237. McGroarty, Patrick and D. Hinshaw. The year ahead: scientists rush to find treatments for Ebola. Jan 2, 2015. A8.
238. Kamp, Jon. Possible Ebola patient transferred to Nebraska. Jan 5, 2015. A2.
239. Loftus, Peter. Ebola drug study to begin. Jan 17, 2015. B3.
240. Burton, Thomas. NIH to test Ebola vaccine with Glaxo and Merck in Liberia. Jan 23, 2015. B3.
241. Loftus, Peter. Chimerix ends testing of Ebola antiviral in Liberia. Feb 2, 2015. B6.
242. Nissenbaum, Dion and J. Barnes. Pentagon to end Ebola mission. Feb 11, 2015. A7.
243. Fujikawa, Megumi. Fujifilm readies Ebola drug. Mar 3, 2015. B7.
244. Bendavid, Naftali. West African leaders warily hail progress on Ebola. Mar 4, 2015. A14.
245. Hinshaw, Drew and A. Morse. Liberia’s last Ebola patient released. Mar 6, 2015. A8.
246. McWhirter, Cameron and B. McKay. Ebola patients find lifeline in special jet. Mar 14, 2015. A3.
247. Calvert, Scott. Ten US Ebola aides exposed. Mar 16, 2015. A6.
248. McKay, Betsy. Ebola proves persistent in Guinea, where crisis started. Apr 2, 2015. A10.
249. Burton, Thomas. Quest for Ebola vaccine beset by test disputes. Apr 10, 2015. A5.
250. Loftus, Peter. A biotech sprouts in Iowa, but can it thrive there? Apr 13, 2015. B1.
251. McKay, Betsy. Health woes plague Ebola survivors. May 1, 2015. A7.
252. Roland, Denise. WHO criticized for initial Ebola response. May 12, 2015. A14.
253. Burton, Thomas. Disputes emerge on African Ebola drug trials. May 13, 2015. A7.
254. Wexler, Alexandra. Foreign fears take toll on African tourism. May 22, 2015. A8.
255. Kieny, Marie-Paule. Testing Ebola treatments ethically. May 26, 2015. A12.
256. McKay, Betsy. Ebola casts a long shadow. Jun 5, 2015. A1.
257. Loftus, Peter. Ebola drug trial is suspended. Jun 20, 2015. B4.
258. Burton, Thomas and P. Loftus. Trials of Ebola vaccine raise hopes. Aug 1, 2015. A6.
259. Hinshaw, Drew. With Ebola in check, Guinea votes. Oct 12, 2015. A9.
260. West, Melanie Grayce. Ebola survivor unites with his caregivers. Oct 21, 2015. A17.
261. Haddon, Heather. Nurse sues over Ebola quarantine. Oct 23, 2015. A17.
262. McKay, Betsy. Ebola failures spark calls for changes. Nov 23, 2015. A13.
263. Hinshaw, Drew and B. McKay. In Liberia, a killer virus won’t stay dead. Dec 10, 2015. A10.
264. Kessel, Mark. Fast diagnosis is needed to prevent Ebola and Zika. Jan 29, 2016. A10.
265. Hinshaw, Drew. Mental woes follow Ebola. Feb 10, 2016. A9.
266. Gottlieb, Scott. Applying to Zika the forgotten lessons of Ebola. Feb 10, 2016. A13.
267. Armour, Stephanie. White House shifts Ebola funds to Zika. Apr 7, 2016. A3.
268. Kanno-Youngs, Zolan. Blue streaks to fight Ebola. May 27, 2016. A14.

**Blogs (All accessed June 14, 2016):**

*CBS.com*

1. CBS News. 35 hospitals designated for Ebola care. Dec 2, 2014. <http://www.cbsnews.com/news/ebola-35-us-hospitals-designated-for-ebola-treatment/>
2. Cooper, Anderson. 60 Minutes investigates medical gear sold during Ebola crisis. May 1, 2016. <http://www.cbsnews.com/news/60-minutes-investigates-medical-gear-sold-during-ebola-crisis/>
3. CBS News. American healthcare worker diagnosed with Ebola, heads to Maryland hospital. Mar 12, 2015. <http://www.cbsnews.com/news/american-healthcare-worker-with-ebola-heads-to-maryland-hospital/>
4. Miller, Jake. As US troops head home, Obama marks “next phase” of Ebola fight. Feb 11, 2015. <http://www.cbsnews.com/news/ebola-fight-obama-marks-next-phase/>
5. Associated Press. UK Nurse who kicked Ebola back in hospital months later. Oct 9, 2015. <http://www.cbsnews.com/news/british-nurse-ebola-hospitalized-complication-serious-condition/>
6. CBS News. CDC chief says US must “rethink” how it handles Ebola. Oct 13, 2014. <http://www.cbsnews.com/news/cdc-chief-says-u-s-must-rethink-how-it-handles-ebola/>
7. Firger, Jessica. Ebola death toll rises in West Africa while Americans’ interest wanes. Jan 8, 2015. <http://www.cbsnews.com/news/ebola-death-toll-rises-in-west-africa-while-us-interest-wanes/>
8. CBS News. Ebola in the USA What you need to know now. <http://www.cbsnews.com/news/ebola-virus-in-us-what-you-need-to-know-now/>
9. CBS News. Doctor infected with Ebola in Sierra Leone to be treated in US. Nov 13, 2014. <http://www.cbsnews.com/news/ebola-outbreak-doctor-infected-in-sierra-leone-to-be-treated-in-omaha-nebraska/>
10. CBS News. Ebola patient Dr. Martin Salia dies in Omaha. Nov 17, 2014. <http://www.cbsnews.com/news/ebola-patient-dr-martin-salia-dies-in-omaha/>
11. Associated Press. Ebola is claiming lives again in Liberia. Jul 15, 2015. <http://www.cbsnews.com/news/ebola-resurgence-liberia-kills-second-person/>
12. CBS News. Ebola survival rate improving in Sierra Leone. Dec 25, 2014. <http://www.cbsnews.com/news/ebola-survival-improving-in-sierra-leone/>
13. Associated Press. Ebola survivors suffer lingering health problems. Aug 24, 2015. <http://www.cbsnews.com/news/ebola-survivors-suffer-lingering-health-problems/>
14. CBS News. Ebola virus can stick around up to 9 months in men. Oct 15, 2015. [http://www.cbsnews.com/news/ebola-virus-can-stick-around-up-to-9-months-in-men/](http://www.cbsnews.com/news/ebola-virus-can-stick-around-up-to-9-months-in-men)
15. CBS News. Ebola virus found lurking in doctor’s eye. May 8, 2015. <http://www.cbsnews.com/news/ebola-virus-found-lurking-in-doctors-eye/>
16. CBS News. Treating Ebola: Inside the first US diagnosis. Oct 26, 2014. <http://www.cbsnews.com/news/ebola-inside-the-first-united-states-diagnosis-thomas-eric-duncan/>
17. Associated Press. Expert hunt origin of new Ebola case in Sierra Leone. Aug 31, 2015. <http://www.cbsnews.com/news/experts-hunt-origin-of-new-ebola-case-in-sierra-leone/>
18. Associated Press. Investigation: US company Metabiota bungled Ebola response. Mar 7, 2016. <http://www.cbsnews.com/news/american-company-metabiota-problems-during-ebola-outbreak/>
19. CBS News. Nurse quarantined in Ebola scare sues New Jersey. Oct 22, 2015. <http://www.cbsnews.com/news/nurse-quarantined-in-ebola-scare-sues-new-jersey/>
20. Patta, Debora. No Christmas for Ebola-ravaged Sierra Leone. Dec 25, 2014. <http://www.cbsnews.com/news/no-christmas-for-ebola-ravaged-sierra-leone/>
21. CBS News. Nurse critically ill from delayed Ebola complications. Oct 14, 2015. <http://www.cbsnews.com/news/delayed-ebola-complications-leave-nurse-critically-ill/>
22. Taylor, Ashley. Possible case of Ebola relapse raises questions. Oct 21, 2015. <http://www.cbsnews.com/news/possible-case-of-ebola-relapse-raises-questions/>
23. Associated Press. Sierra Leone loses its 10^th^ doctor to Ebola outbreak. Dec 7, 2014. <http://www.cbsnews.com/news/sierra-leone-loses-its-10th-doctor-to-ebola-outbreak/>
24. CBS News. The Ebola Hot Zone. Nov 9, 2014. <http://www.cbsnews.com/news/the-ebola-hot-zone-liberia/>
25. CBS News. WHO declares official end to Ebola outbreak in West Africa. Jan 14, 2016. <http://www.cbsnews.com/news/who-declares-official-end-to-ebola-outbreak-in-west-africa/>
26. CBS News. World “on the verge of an effective Ebola vaccine”. Jul 31, 2015. <http://www.cbsnews.com/news/who-ebola-vaccine-trial-in-guinea-a-game-changer/>
27. Schupak, Amanda. Chimps and gorillas are Ebola’s unseen victims. Jan 22, 2015. <http://www.cbsnews.com/news/chimps-gorillas-ebola-unseen-victims/>
28. Dallas, Mary Elizabeth. Rapid-detection Ebola test could be game-changer. <http://www.cbsnews.com/news/sierra-leone-loses-its-10th-doctor-to-ebola-outbreak/>
29. Associated Press. More than 10,000 people infected with Ebola, WHO says. Oct 25, 2014. <http://www.cbsnews.com/news/ebola-outbreak-more-than-10000-people-infected-who-says/>

*NBC.com*

1. Fox, Maggie. A Good Warrior: Newborn Beast Ebola in Guinea. Nov 22, 2015. <http://www.nbcnews.com/storyline/ebola-virus-outbreak/good-warrior-newborn-beats-ebola-guinea-n467331>
2. Fox, Maggie. American Infected With Ebola Returning for Treatment. Mar 12, 2015. <http://www.nbcnews.com/storyline/ebola-virus-outbreak/another-american-infected-ebola-n322486>
3. Fox, Maggie. British Nurse Better After Ebola Complications Cause Meningitis. Oct 21, 2015. <http://www.nbcnews.com/storyline/ebola-virus-outbreak/british-nurse-better-after-ebola-complications-cause-meningitis-n448511>
4. Fox, Maggie. Could Sex Have Brought Ebola Back to Liberia? Mar 25, 2015. <http://www.nbcnews.com/storyline/ebola-virus-outbreak/could-sex-have-brought-ebola-back-liberia-n330071>
5. Fox, Maggie. Ebola Cases Rise Again in West Africa. Jun 10, 2015. <http://www.nbcnews.com/storyline/ebola-virus-outbreak/ebola-ticks-again-west-africa-n373171>
6. Fox, Maggie. Ebola Epidemic Slows Even More, World Health Organization Says. May 13, 2015. <http://www.nbcnews.com/storyline/ebola-virus-outbreak/ebola-epidemic-slows-even-more-n358376>
7. Fox, Maggie. Ebola is Still Lurking, and Killing, WHO Says. Jul 15, 2015. <http://www.nbcnews.com/storyline/ebola-virus-outbreak/ebola-still-lurking-killing-who-says-n392671>
8. Fox, Maggie. Ebola Lingers in Semen for Months, Studies Confirm. Oct 15, 2015. <http://www.nbcnews.com/storyline/ebola-virus-outbreak/ebola-lingers-semen-months-studies-confirm-n444736>
9. Fox, Maggie. Ebola Quarantines Were Stupid and Wrong, Report Says. Dec 3, 2015. <http://www.nbcnews.com/storyline/ebola-virus-outbreak/ebola-quarantines-were-stupid-wrong-report-says-n473596>
10. Fox, Maggie. Ebola Rates Plummet, But WHO Says More to Come. Aug 5, 2015. <http://www.nbcnews.com/storyline/ebola-virus-outbreak/ebola-rates-plummet-who-says-more-come-n404696>
11. Fox, Maggie. Ebola Returns to Liberia, Again. Nov 23, 2015. <http://www.nbcnews.com/storyline/ebola-virus-outbreak/ebola-returns-liberia-again-n467146>
12. Fox, Maggie. Ebola Survivor Back in Hospital Suffers Lingering Hold of Virus. Oct 10, 2015. <http://www.nbcnews.com/storyline/ebola-virus-outbreak/ebola-survivor-back-hospital-suffers-lingering-hold-virus-n441761>
13. Fox, Maggie. Ebola Survivors Suffer Long-Term Consequences: Studies. Feb 24, 2016. <http://www.nbcnews.com/storyline/ebola-virus-outbreak/ebola-survivors-suffer-long-term-consequences-studies-n525146>
14. Fox, Maggie. Ebola’s Back, Just Hours After West Africa Hits Zero. Jan 15, 2016. <http://www.nbcnews.com/storyline/ebola-virus-outbreak/ebola-s-back-just-hours-west-africa-hits-zero-n497321>
15. Fox, Maggie. Experimental Ebola Shot Shows Good Response. Apr 1, 2015. <http://www.nbcnews.com/storyline/ebola-virus-outbreak/experimental-ebola-shot-shows-good-response-n334056>
16. Associated Press. Experimental Ebola Vaccine Could Stop Virus. Jul 13, 2015. <http://www.nbcnews.com/storyline/ebola-virus-outbreak/experimental-ebola-vaccine-could-stop-virus-n401586>
17. Reuters. Fifth Person Dies as Ebola Comes Back to Guinea. Mar 22, 2016. <http://www.nbcnews.com/storyline/ebola-virus-outbreak/fifth-person-dies-ebola-comes-back-guinea-n543721>
18. Reuters. Health Workers Head in to Contain Fresh Ebola Outbreak in Guinea. Mar 18, 2016. <http://www.nbcnews.com/storyline/ebola-virus-outbreak/health-workers-head-contain-fresh-ebola-outbreak-guinea-n541411>
19. Fox, Maggie. Horror in Sierra Leone: A Single Spark Gives Ebola New Life. Dec 13, 2014. <http://www.nbcnews.com/storyline/ebola-virus-outbreak/horror-sierra-leone-single-spark-gives-ebola-new-life-n267421>
20. Johnson, Alex. Kaci Hickox, Maine Nurse Quarantined in Ebola Scare, Sues New Jersey Gov. Chris Christie. Oct 22, 2015. <http://www.nbcnews.com/storyline/ebola-virus-outbreak/kaci-hickox-maine-nurse-quarantined-ebola-scare-sues-new-jersey-n449491>
21. Jamieson, Alastair and D. Wyllie. Liberia is Free of Ebola, says World Health Organization. May 9, 2015. <http://www.nbcnews.com/storyline/ebola-virus-outbreak/liberia-free-ebola-says-world-health-organization-n356441>
22. NBC News. Liberia Reports Second Case of Ebola. <http://www.nbcnews.com/health/health-news/liberia-reports-second-case-ebola-n385116>
23. Fox, Maggie. Liberia’s Ebola-Free, But Not Out of the Woods Yet. May 12, 2015. <http://www.nbcnews.com/storyline/ebola-virus-outbreak/liberias-ebola-free-not-out-woods-yet-n357301>
24. Fox, Maggie. Pauline Cafferkey, Nurse With Ebola Complications, is ‘Critically Ill’. Oct 14, 2015. <http://www.nbcnews.com/storyline/ebola-virus-outbreak/pauline-cafferkey-nurse-ebola-complications-critically-ill-n444371>
25. Fox, Maggie. Teenaged Boy Dies of Ebola in Liberia. Nov 24, 2015. <http://www.nbcnews.com/health/health-news/teenaged-boy-dies-ebola-liberia-n468876>
26. Fox, Maggie. The Next Ebola Zone: Report Finds 28 High-Risk Countries. Mar 2, 2015. <http://www.nbcnews.com/storyline/ebola-virus-outbreak/next-ebola-zone-report-finds-28-high-risk-countries-n316436>
27. Reuters. Two Diagnosed With Ebola in Guinea. Mar 17, 2016. <http://www.nbcnews.com/storyline/ebola-virus-outbreak/two-diagnosed-ebola-guinea-n541056>
28. Fox, Maggie. Vaccine, Not Ebola, Made Doc Sick, Study Finds. Mar 5, 2015. <http://www.nbcnews.com/storyline/ebola-virus-outbreak/vaccine-not-ebola-made-doc-sick-n318131>
29. Fox, Maggie. West Africa Counts Down to Possible End of Ebola, World Health Organization Says. Nov 11, 2015. <http://www.nbcnews.com/storyline/ebola-virus-outbreak/vaccine-not-ebola-made-doc-sick-n318131>
30. Associated Press. WHO: Ebola Outbreak is West Africa Is Over. Jan 14, 2016. <http://www.nbcnews.com/storyline/ebola-virus-outbreak/who-ebola-outbreak-west-africa-over-n496306>

*Yahoo!/ABC.com*

1. Associated Press. After Ebola, World Bank creates pandemic insurance plan. May 21, 2016. <https://www.yahoo.com/news/ebola-world-bank-creates-pandemic-insurance-plan-032206770--finance.html>
2. Reuters. Boy tests positive for Ebola in latest Liberia flare-up. Apr 3, 2016. <https://www.yahoo.com/news/boy-tests-positive-ebola-latest-liberia-flare-210508128.html>
3. Associated Press. Ebola claims another victim in Guinea as vaccinations ramped up. Apr 5, 2016. <https://www.yahoo.com/news/ebola-claims-another-victim-guinea-vaccinations-ramped-192323781.html>
4. Quartz. Ebola resurgences in West Africa suggest the virus can linger longer than expected. Apr 24, 2016. <http://finance.yahoo.com/news/ebola-resurgences-west-africa-suggest-152742720.html>
5. Market wired. GeoVax Ebola Vaccine Protects Non-Human Primates Against Lethal Challenge. Jun 1, 2016. <http://finance.yahoo.com/news/geovax-ebola-vaccine-protects-non-130000610.html>
6. Reuters. Guines declared free of active Ebola transmission: WHO. Jun 1, 2016. <https://www.yahoo.com/news/guinea-declared-free-active-ebola-transmission-083505125.html>
7. Reuters. Guinea seeks rebound from Ebola with double-digit growth by 2020. May 4, 2016. <https://www.yahoo.com/news/guinea-seeks-rebound-ebola-double-digit-growth-2020-003407264--business.html>
8. Associated Press. Last known Ebola patient discharged in Guinea. Apr 20, 2016. <https://www.yahoo.com/news/last-known-ebola-patient-discharged-guinea-183522741.html>
9. Associated Press. Latest Ebola outbreak in Guinea is over: WHO. Jun 1, 2016. <https://www.yahoo.com/news/latest-ebola-outbreak-guinea-over-081529709.html>
10. Associated Press. Liberia confirms 2^nd^ new Ebola care, possibly from Guinea. Apr 3, 2016. <https://www.yahoo.com/news/once-ebola-free-liberia-confirms-2nd-case-101918629.html>
11. Associated Press. Liberia Ebola outbreak to be declared over. Jun 8, 2016. <https://www.yahoo.com/news/liberia-ebola-outbreak-declared-over-025922136.html>
12. Associated Press. Liberia’s last known Ebola patients discharged from hospital. May 4, 2016. <https://www.yahoo.com/news/liberias-last-known-ebola-patients-discharged-hospital-154545570.html>
13. PR Newswire. New Idylla Ebola virus triage test granted emergency use authorization by US FDA. Jun 1, 2016. <http://finance.yahoo.com/news/idylla-ebola-virus-triage-test-050000082.html>
14. Associated Press. One known Ebola case left in Guinea after girl’s discharge. Apr 14, 2016. <https://www.yahoo.com/news/one-known-ebola-case-left-guinea-girls-discharge-155254628.html>
15. Associated Press. Sierra Leone Ebola survivors protest government inaction. Apr 4, 2016. <https://www.yahoo.com/news/sierra-leone-ebola-survivors-protest-government-inaction-201952244.html>
16. Associated Press. Sierra Leone on alter after new W. Africa Ebola cases. Apr 10, 2016. <https://www.yahoo.com/news/sierra-leone-alert-w-africa-ebola-cases-175105295.html>
17. O’Mahony, Jennifer. Sierra Leone’s first Ebola-hit community reconsiders its traditions. May 12, 2016. <https://www.yahoo.com/news/sierra-leones-first-ebola-hit-community-reconsiders-traditions-062044848.html>
18. Reuters. Study of Liberia Ebola flare-up shows need for longer vigilance. Apr 30, 2016. <https://www.yahoo.com/news/study-liberia-ebola-flare-shows-longer-vigilance-090415027.html>
19. Associated Press. Teenage boy dies of Ebola in Liberia after months without new cases. Nov 24, 2015. <http://www.huffingtonpost.com/entry/ebola-death-liberia_us_565487b9e4b0879a5b0c704f>
20. Associated Press. Latest Ebola outbreak in Guinea is now over. Jun 1, 2016. <https://www.yahoo.com/news/un-latest-ebola-outbreak-guinea-now-over-145537081.html>
21. Dosso, Zoom. West Africa marks end of deadly Ebola outbreak. Jun 9, 2016. <https://www.yahoo.com/news/ebola-outbreak-over-says-liberia-health-ministry-092053070.html>
22. Associated Press. White House: $589M from Ebola to go to fight Zika virus. Apr 6, 2016. <https://www.yahoo.com/news/white-house-transfer-ebola-funds-combat-zika-virus-041010372.html>
23. Reuters. WHO declares Liberia free of active Ebola virus transmission. Jun 9, 2016. <https://www.yahoo.com/news/declares-liberia-free-active-ebola-virus-transmission-105324958.html>

*Huffington Post*

1. Toweh, Alphonso. Liberia suffer new Ebola death months after outbreak declared over. Apr 1, 2016, <http://www.huffingtonpost.com/entry/liberia-new-ebola-death_us_56fe7312e4b0daf53aef62ad>?
2. Rose, Joan. 2014: The Year of Ebola. Jan 13, 2015. <http://www.huffingtonpost.com/joan-b-rose/2014-the-year-of-the-viru_b_6459052.html>
3. McLaughlin, Michael. After 2 years and 2,500 deaths, Guinea is Ebola-Free. Dec 29, 2015. <http://www.huffingtonpost.com/entry/guinea-ebola-free_us_5671aa88e4b0648fe301d3e1>
4. Weintraub, Karen and L. Szabo. American Ebola survivor Kent Brantly donates blood to fellow missionary doctor. Sep 14, 2014. <http://www.huffingtonpost.com/2014/09/14/ebola-survivor-donates-blood_n_5813772.html>
5. Goldberg, Eleanor. As Ebola outbreak spreads, here’s how you can help. Sep 16, 2014. <http://www.huffingtonpost.com/2014/09/16/help-ebola-victims_n_5830060.html>
6. Delaney, Arthur. CDC removed info on coughing and sneezing from Ebola Q&A (Update). Oct 30, 2014. <http://www.huffingtonpost.com/2014/10/30/cdc-ebola_n_6078072.html>
7. Bobic, Igor. Chris Christie to nurse who may sue him: ‘Whatever. Get in line’. Oct 29, 2014. <http://www.huffingtonpost.com/2014/10/29/chris-christie-ebola-nurse-whatever_n_6067342.html>
8. Nzwill, Frederick. Church leaders claim new band aid song reinforces negative stereotypes about Africa. Dec 11, 2014. <http://www.huffingtonpost.com/2014/12/11/band-aid-africa-_n_6291068.html>
9. King, Christopher. Despite headlines, Ebola doesn’t yet register strongly in the scientific literature. Sep 18, 2014. <http://www.huffingtonpost.com/christopher-king/despite-headlines-ebola-d_b_5835326.html>
10. Horowitz, Alana and A. Almendrala. Ebola diagnosed in US for the first time: CDC. Sep 30, 2014. <http://www.huffingtonpost.com/2014/09/30/ebola-us-cdc_n_5909394.html>
11. Stein, Sam and A. Delaney. Ebola is nearly gone and largely forgotten. Thank the government, says Ebola czar. May 11, 2015. <http://www.huffingtonpost.com/2015/05/11/ebola-ron-klain_n_7259436.html>
12. Manning, Nathaniel. Ebola is not the only story about Africa: an African technological renaissance. Oct 17, 2014. <http://www.huffingtonpost.com/nathaniel-manning/ebola-is-not-the-only-sto_b_6001498.html>
13. Stein, Sam. Ebola vaccine would likely have been found by now if not for budget cuts NIH director. Oct 12, 2014. <http://www.huffingtonpost.com/2014/10/12/ebola-vaccine_n_5974148.html>
14. Brown, Jennifer. Everything you need to know about Ebola and your pets. Oct 21, 2014. <http://www.huffingtonpost.com/2014/10/21/ebola-pets_n_6015084.html>
15. McLaughlin, Michael. Finally, the deadliest Ebola outbreak is almost over. Dec 29, 2015. <http://www.huffingtonpost.com/entry/ebola-outbreak-2015_us_5682ee8ee4b06fa6888155fe>
16. Brooks, Rob. How often does Donald Trump wash his hands? Apr 4, 2016. <http://www.huffingtonpost.com/rob-brooks/how-often-does-donald-tru_b_9607218.html>
17. Chambers, Ray. In conversation: David Nabarro, the man on the front line of Ebola crisis. Sep 16, 2014. <http://www.huffingtonpost.com/ray-chambers/in-conversation-david-nab_b_5829246.html>
18. Siddiqui, Sabrina. Kay Hagan: Obama should ‘Immediately’ impose Ebola flight ban. Oct 17, 2014. <http://www.huffingtonpost.com/2014/10/17/kay-hagan-ebola_n_6005078.html>
19. Reuters. New cases of Ebola found in Liberia. Nov 20, 2015. <http://www.huffingtonpost.com/entry/ebola-liberia_us_564f2981e4b0258edb30f9bb>
20. Sieczkowski, Cavan. No, a ‘sexy Ebola containment suit’ Halloween costume is NOT OK. Oct 27, 2014. <http://www.huffingtonpost.com/2014/10/27/sexy-ebola-containment-suit-halloween_n_6054068.html>
21. Stein, Sam and Z. Carter. Obama also pushed for CDC cuts in years before Ebola outbreak. Oct 15, 2014. <http://www.huffingtonpost.com/2014/10/15/obama-cdc-funding_n_5990114.html>
22. Bobic, Igor. Republican who wants West Africa flight ban unaware there are no direct flights to US. Oct 23, 2014. <http://www.huffingtonpost.com/2014/10/17/dennis-ross-ebola-flight-ban_n_6002730.html>
23. Chan, Amanda. Striking photos of the worst Ebola outbreak in history. July 29, 2014. <http://www.huffingtonpost.com/2014/07/29/ebola-outbreak-west-africa-photos-virus_n_5631405.html>
24. Robins-Early, Nick. The Ebola outbreak started way earlier than you thought. Aug 29, 2014. <http://www.huffingtonpost.com/2014/08/29/ebola-outbreak_n_5730784.html>
25. Almendrala, Anna. The most destructive myths about Ebola virus, debunked. Aug 6, 2014. <http://www.huffingtonpost.com/2014/08/06/ebola-myths_n_5655662.html>
26. Chan, Amanda. These photos reveal the grim reality of Ebola in Sierra Leone. Sep 23, 2014. <http://www.huffingtonpost.com/2014/09/23/tommy-trenchard-ebola-sierra-leone-liberia_n_5851314.html>
27. Smith, Raymond. Time’s ‘Person of the Year’ spotlights the crucial role of Ebola survivors. Dec 11, 2014. <http://www.huffingtonpost.com/raymond-a-smith-phd/times-person-of-the-year-_2_b_6309798.html>
28. Kuruvilla, Carol. What are America’s religious organizations doing to prepare for Ebola? Oct 17, 2014. <http://www.huffingtonpost.com/2014/10/17/america-church-ebola_n_6003344.html>
29. Weizel, Richard. Yale University student being tested for Ebola. Oct 16, 2014. <http://www.huffingtonpost.com/2014/10/16/yale-student-ebola_n_5998918.html>

*CNN.com*

1. Cohen, Elizabeth. 1 dead after Ebola reappears in Liberia. Jun 30, 2015. <http://www.cnn.com/2015/06/30/health/new-ebola-case-liberia/>
2. Cohen, Elizabeth and S. Almasy. Americans exposed to Ebola return for monitoring. Mar 15, 2015. <http://www.cnn.com/2015/03/14/health/cdc-americans-ebola/>
3. Ihekeweazu, Chikwe. Beating Ebola? It’s a matter of trust. May 8, 2015. <http://www.cnn.com/2015/05/08/opinions/the-cause-of-ebola-in-liberia/>
4. Cullinane, Susannah and N. Thompson. Deadliest ever outbreak of Ebola virus: What to known. Sep 18, 2014. <http://www.cnn.com/2014/03/27/world/ebola-virus-explainer/>
5. Smith-Spark, Laura. WHO: New Ebola cases drop to lowest level in 7 months. Jan 29, 2015. <http://www.cnn.com/2015/01/29/africa/ebola-virus/>
6. Ap, Tiffany. Ebola crisis: WHO slammed by Harvard-convened panel over slow response. Nov 23, 2015. <http://www.cnn.com/2015/11/23/africa/ebola-lancet-report/>
7. CNN Library. Ebola Fast Facts. Apr 22, 2016. <http://www.cnn.com/2014/04/11/health/ebola-fast-facts>
8. Karimi, Faith and D. McKenzie. Ebola resurfaces in Sierra Leone hours after WHO declares outbreak over. Jan 15, 2016. <http://www.cnn.com/2016/01/15/africa/sierra-leone-ebola-new-case/>
9. Cohen, Elizabeth. New Ebola treatments mostly failing. Jan 11, 2016. <http://www.cnn.com/2016/01/11/health/ebola-hiv-drugs-update/>
10. Smith-Spark, Laura. WHO: Trials show new Ebola vaccine is ‘highly effective’. Aug 3, 2015. <http://www.cnn.com/2015/07/31/health/guinea-ebola-vaccine/>
11. Cullinane, Susannah and M. Park. Ebola virus: nine things to know about the killer disease. Jul 31, 2015. <http://www.cnn.com/2014/08/07/world/ebola-virus-q-and-a/>
12. Hanna, Jason and R. Coleman. Sierra Leone has 2^nd^ Ebola case after outbreak’s end. Jan 21, 2016. <http://www.cnn.com/2016/01/21/africa/sierra-leone-ebola/>
13. Cohen, Elizabeth. Fighting Ebola with HIV drug gets big shot in the arm. May 8, 2015. <http://www.cnn.com/2015/05/01/health/ebola-hiv-research/>
14. Melvin, Don. Guinea declared free of Ebola virus. Dec 29, 2015. <http://www.cnn.com/2015/12/29/africa/guinea-end-of-ebola/>
15. Nurse, Earl and P. Guest. How Sierra Leone plans to bounce back after Ebola. Dec 23, 2015. <http://www.cnn.com/2015/11/12/africa/sierra-leone-ebola-recovery/>
16. Christensen, Jen. Lesson learned after Ebola. Oct 15, 2015. <http://www.cnn.com/2015/04/14/health/ebola-1-year-later/>
17. Hanna, Jason. Liberia once again Ebola-free, WHO says. Sep 3, 2015. <http://www.cnn.com/2015/09/03/health/liberia-ebola/>
18. Smith-Spark, Laura. Spain declared free of Ebola virus by WHO. Dec 3, 2014. <http://www.cnn.com/2014/12/02/world/europe/spain-ebola-free/>
19. Wilson, Jacque. US Ebola survivors: Where are they now? Sep 2, 2015. <http://www.cnn.com/2015/09/02/health/ebola-outbreak-survivors-rewind/>
20. Hume, Tim. UK’s first Ebola patient back in hospital in serious condition after complication. Oct 9, 2015. <http://www.cnn.com/2015/10/09/europe/uk-ebola-nurse/>
21. Botelho, Greg. 9 months after discharge, relapsed UK Ebola patient ‘critically ill’. Oct 15, 2015. <http://www.cnn.com/2015/10/14/europe/uk-ebola-nurse/>
22. Melvin, Don. WHO: Ebola virus found in male survivors 9 months after symptoms. Oct 15, 2015. <http://www.cnn.com/2015/10/15/health/who-ebola-report/>

**Alternative Sites:**

*InfoWars.com*

1. Daniels, Kit. 85% of Nurses Not Trained For Ebola. Oct 13, 2014. <https://www.infowars.com/85-of-nurses-not-trained-for-ebola/>
2. Nimmo, Kurt. CDC tells airlines ‘treat all body fluid as infectious’. September 21, 2014. <https://www.infowars.com/cdc-tells-airlines-treat-all-body-fluids-as-infectious/>
3. RT. DARPA biotech division seeks ideas to solve Ebola crisis, prepare for ‘next thing’. Nov 8, 2014. <https://www.infowars.com/darpa-biotech-division-seeks-ideas-to-solve-ebola-crisis-prepare-for-next-thing/>
4. Belluz, Julia. Ebola Czar – “This Thing Isn’t Over Yet”. And the next pandemic could be eeven worse. March 3, 2015. <https://www.infowars.com/ebola-czar-this-thing-isnt-over-yet-and-the-next-pandemic-could-be-even-worse>
5. Hodges, David. How Ebola Will Irreversibly Transform America. September 23, 2014. <https://www.infowars.com/how-ebola-will-irreversibly-transform-america/>
6. Perry, Jack. The Great Father’s Ebola Solution: More Government Corruption. Oct 19, 2014. <https://www.infowars.com/the-great-fathers-ebola-solution-more-government-corruption/>

*VeteransToday.com*

1. In 2014, there was the western Africa Ebola hoax. March 24, 2015.
2. Heart, Jack. The Blood of Christ – Hemorrhagic Fever, Expendable Humans and Bacteria Gone BeZerk (sic)… March 2016.

*Nodisinfo.com*

1. Dallas Ebola Scare is an arch-Zionist Hoax. Oct 3, 2014.

*NewsBusters.com*

1. Meyer, Jeffrey. ABC’s Stephanopoulos rushes to defend Obama’s Ebola Czar. Oct 19, 2014.
2. Drennen, Kyle. NBC Medical editor violates Ebola Quarantine, dodges responsibility. Oct 14, 2014.
3. Finkelstein, Mark. Obama-Defending Doc Snaps at Nicolle Wallace: ‘Stick with Netflix’ for Ebola ‘hysteria’. Oct 16, 2014.
4. Baker, Brent. SNL ridicules Obama and his Ebola Czar Ron Klain. Oct 26, 2014.

*Beforeitsnews.com*

1. Mandatory Ebola Vaccines. Oct 10, 2014. <http://beforeitsnews.com/alternative/2014/10/alert-shtf-is-here-news-the-government-does-not-want-you-to-know-connecticut-governor-mandatory-ebola-vaccines-vic>
2. Brown, Tim. West Africans are streaming across the US Southern border carrying the Ebola virus. Aug 4, 2014. <http://beforeitsnews.com/alternative/2014/08/west-africans-are-streaming-across-the-u-s-southern-border-carrying-the-ebola-virus-3006056.html>
3. The US patented the Ebola virus just as the failure of the false flag swine flu pandemic became apparent. Oct 2, 2014. <http://beforeitsnews.com/power-elite/2014/10/the-us-patented-the-ebola-virus-just-as-the-failure-of-the-false-flag-swine-flu-pandemic-became-apparent-2446964.html>
4. Should you worry about an Ebola outbreak in the US? August 19, 2014. <http://beforeitsnews.com/healthcare/2014/08/should-you-worry-about-an-ebola-outbreak-in-the-us-2457894.html>
5. Researchers: Can’t Stop Ebola Without Travel Ban. Oct 22, 2014. <http://beforeitsnews.com/alternative/2014/10/researchers-cant-stop-ebola-without-travel-ban-3049578.html>
6. Plainclothes man at Ebola Scene perplexes viewers. Oct 15, 2014. <http://beforeitsnews.com/alternative/2014/10/plainclothes-man-at-ebola-scene-perplexes-viewers-3045724.html>
7. Biohoax – Ebola US health dept epidemiologist states “the public is not at risk … transmission is difficult”. August 20, 2014. <http://beforeitsnews.com/healthcare/2014/08/biohoax-ebola-us-health-dept-epidemiologist-states-the-public-is-not-at-risk-transmission-is-difficult-north-american-ebol>
8. Biohoax – Now Ebola is NOT in New Mexico. Aug 18, 2014. <http://beforeitsnews.com/healthcare/2014/08/biohoax-article-review-now-ebola-is-not-in-new-mexico-yesterday-it-was-not-in-albania-and-scotland-just-keep-testing-e>
9. Ebola – your’re (sic) sure? You’re real sure? Oct 10, 2014. <http://beforeitsnews.com/opinion-conservative/2014/10/ebola-yourre-sure-youre-real-sure-2918818.html>
10. Dept of Defense Ebola Manual: smoking gun. Oct 19, 2014. <http://beforeitsnews.com/alternative/2014/10/dept-of-defense-ebola-manual-smoking-guns-3-3047482.html>
11. Ebola Virus, 2th October, announced for long LieKiller YT channel. October 7, 2014. <http://beforeitsnews.com/new-world-order/2014/10/ebola-virus-24th-october-announced-for-long-on-liekillers-yt-channel-antichrists-official-tribune-2906.html>
12. Media Blackout: Government plans to place Ebola patients in hotels. April 9, 2015. <http://beforeitsnews.com/blogging-citizen-journalism/2015/04/media-blackout-government-plans-to-place-ebola-patients-in-hotels-2504860.html>
13. Tavernise, Sabrina. Newly vigilant, US will screen fliers for Ebola. Oct 9, 2014. <http://beforeitsnews.com/tea-party/2014/10/newly-vigilant-u-s-will-screen-fliers-for-ebola-by-sabrina-tavernise-2538548.html>
